# β-Aminobutyric Acid Priming Acquisition and Defense Response of Mango Fruit to *Colletotrichum gloeosporioides* Infection Based on Quantitative Proteomics

**DOI:** 10.3390/cells8091029

**Published:** 2019-09-04

**Authors:** Taotao Li, Panhui Fan, Ze Yun, Guoxiang Jiang, Zhengke Zhang, Yueming Jiang

**Affiliations:** 1 Key Laboratory of Plant Resources Conservation and Sustainable Utilization/Guangdong Provincial Key Laboratory of Applied Botany, South China Botanical Garden, Chinese Academy of Sciences, Guangzhou 510650, China (T.L.) (Z.Y.) (G.J.) (Y.J.); 2 College of Food Science and Engineering, Hainan University, Haikou 570228, China

**Keywords:** stress, fruit, priming, β-aminobutyric acid, anthracnose, proteome

## Abstract

β-aminobutyric acid (BABA) is a new environmentally friendly agent to induce disease resistance by priming of defense in plants. However, molecular mechanisms underlying BABA-induced priming defense are not fully understood. Here, comprehensive analysis of priming mechanism of BABA-induced resistance was investigated based on mango-*Colletotrichum gloeosporioides* interaction system using iTRAQ-based proteome approach. Results showed that BABA treatments effectively inhibited the expansion of anthracnose caused by *C. gleosporioides* in mango fruit. Proteomic results revealed that stronger response to pathogen in BABA-primed mango fruit after *C. gleosporioides* inoculation might be attributed to differentially accumulated proteins involved in secondary metabolism, defense signaling and response, transcriptional regulation, protein post-translational modification, etc. Additionally, we testified the involvement of non-specific lipid-transfer protein (nsLTP) in the priming acquisition at early priming stage and memory in BABA-primed mango fruit. Meanwhile, spring effect was found in the primed mango fruit, indicated by inhibition of defense-related proteins at priming phase but stronger activation of defense response when exposure to pathogen compared with non-primed fruit. As an energy-saving strategy, BABA-induced priming might also alter sugar metabolism to provide more backbone for secondary metabolites biosynthesis. In sum, this study provided new clues to elucidate the mechanism of BABA-induced priming defense in harvested fruit.

## 1. Introduction

Defense response is essential during plants life when constantly exposed to stressful situations due to changing environmental conditions, especially when interacting with pathogenic microorganisms. Among sophisticated defense mechanisms of plants, induced resistance through recognizing the presence of a pathogen is also vital for appropriate and in time defenses against attacking pathogens [1]. Priming is one of the inducible defense mechanisms in plants in response to different stresses. The priming process can be divided into two stages: Stimuli-induced “priming phase” that is characterized by the status without the obvious induction of defense; and the “primed stage” that implies the state of activated defense response [2]. During the whole priming events, slight alteration of various primary and secondary metabolites, enzymes, hormones, and signal molecules can occur in the priming stage, which put the plant in a standby state [3]. Once affected by these stresses, plants enters the post-challenge primed state and responds more rapidly and or more efficiently to such stresses [3,4]. Compared with direct activation of defense response, priming-induced defense response can be considered as a kind of cost-effective behavior in evolutional disease management of plants. Wang et al. [5] suggested that the priming defense appeared as an optimal strategy that balances disease protection and maintenance of sensory quality in postharvest fruit during storage. Meanwhile, defense of priming has now emerged as a promising means for sustainable modern management of crops due to its practical availability in improving resistance without influence on yields [2,4]. Hence, the research elucidating the molecular mechanisms of priming is vital for guiding wider application of priming on crops. During the past decades, priming has been proved as a critical process in plant immunity, advancing our understanding on defense response of higher plants. Nevertheless, identification of more hypothetical genes and proteins is still essential for thorough understanding of the detailed priming defense mechanisms.

β-Aminobutyric acid (BABA) is a non-proteinogenic amino acid that was identified as inducer of disease resistance in 1963 [6]. BABA has been reported to protect a large number of plant species against diverse pathogens and pests, making an outstanding contribution to stabilizing yield of crops [3,6,7]. In the last 20 years, numerous studies also reveal that BABA can prime defense responses to fungal pathogens on a range of postharvest fruits, including citrus [8], apple [9], mango [10], grape berries [11], strawberries [5], and peaches [12]. Unfortunately, most research on priming defense against postharvest diseases has only concentrated on the investigation for physiological parameters and expression profiling of key genes during the post-challenge primed state. Thereby, it is imperative to reveal molecular mechanisms of BABA-induced resistance against pathogens by priming in postharvest fruit. Though molecular mechanisms of BABA-induced resistance have been intensively studied in Arabidopsis [1,7], it might vary in different plant species, especially in crops, including fruit.

Omics-based researches on the plant response to different stresses have been widely conducted, including priming defenses against plant fungal diseases [3]. Importantly, proteomic data from mass spectrometry analysis could also be used to correct and validate protein coding genes and effectively quantify the most valuable protein participants in the organism [13]. Few studies have dissected the BABA-mediated priming defense in plants based on proteomics technology [14,15]. However, these researches mainly deal with post-challenge primed state of entire priming event, but early signaling involved in defense preparation that precede activated defense response are largely unknown. Considering the important roles of proteins in the priming event, comprehensive proteomic investigation of the whole priming induced by BABA is still urgent. Unfortunately, till now, proteomic information revealing the mechanisms of BABA-induced priming defense in mango fruit was lacking. 

Mango (*Mangifera indica* L.) is a favorite tropical fruit due to its pleasant flavor, outstanding nutritional qualities, and high marketing value. However, mango fruit belongs to a typical climacteric fruit and has a relatively short shelf life when stored at ambient temperatures. Moreover, mango fruit are liable to postharvest decay resulting from infection of various pathogens, leading to major economic losses. *Colletotrichum gloeosporioides* (Penz) is well known for its pathogenicity causing anthracnose disease in mango fruit [16]. Till now, fungicides such as benomyl and prochlo-raz have been used to control postharvest anthracnose in mango fruit [16]. Due to the negative impact of fungicides on human health and the environment, some alternative strategies based on abiotic or biotic factors have been developed, such as exogenous treatments with nitric oxide [16], salicylic acid (SA) [17], and oxalic acid [18]. In our previous research, BABA treatment demonstrated a prominent ability to suppress mango anthracnose by an elicitation of priming defense in mango fruit during postharvest storage [10]. Though the increased disease resistance caused by BABA was clear, the changes of mango fruit at early priming stage and the post-challenge defense pathways activated by priming are still not fully understood. Therefore, it has theoretical and practical significance to study the molecular mechanism underlying the BABA priming using high-throughput technologies, particularly for the plant crops without genome information.

In the present study, we investigated the proteomics differences between BABA-primed and non-primed mango fruits in priming stage (pre-inoculation of *C. gloeosporioides*) and post-challenge primed state (post-inoculation of *C. gloeosporioides*) using high-throughput iTRAQ protocol. Accompanied with physiological and RT-qPCR results, we can better understand the molecular mechanisms underlying the priming defense of mango against *C. gloeosporioides* at entire priming scale.

## 2. Materials and Methods

### 2.1. Plant Material and Treatment 

Mango (*Mangifera indica* Linn.) fruit was harvested at mature green stage as shown in our previous research [10] from a commercial orchard located in Dongfang city, Hainan province, China. The fruit were packed and transported to laboratory in an air-conditioned cargo van at 25 °C. Fruit with uniform size, shape, ripeness, and absence of visible symptoms of disease and mechanical injury were selected for the experiments. After disinfecting with 2% (v/v) sodium hypochlorite for 2 min, the fruits were air-dried and divided randomly into four groups. Fruits treated with distilled water containing 0.05% (v/v) Tween 80 for 5 min were designated as Water group (Control); fruits dipped in 50 mM BABA solutions containing 0.05% (v/v) Tween 80 at 25 ± 1 °C for 5 min described in our previous research [10] were designated as BABA group. After 24 h of treatments with BABA or water, the mango fruits were inoculated with *C. gloeosporioides* (1 × 10^6^ spores mL^−1^) according to our previous method [10]. The fruits with inoculation were designated as Water+*C. gloeosporioides* and BABA + *C. gloeosporioides* group, respectively. All fruits were stored at 25 °C, RH 85%–90% in darkness for 8 days. During storage, diameters of lesions in inoculated mango fruit were recorded at 0, 2, 4, 6, and 8 d. Each treatment for lesions investigation had three replicates, with 30 fruit per replicate.

### 2.2. Sample Collecting

Before inoculation, the fruit exocarp tissues were taken at 0, 6, 12, 18, and 24 h during storage. After inoculation, the fruit exocarp tissue was taken at 2, 4, 6, and 8 d during storage. All the tissue samples were frozen in liquid nitrogen and stored at −80 °C before use. Each treatment for sampling included three replicates, with 9 fruit for each replicate.

### 2.3. Measurement of Total Phenolics, Flavonoids, and Lignin Content 

One gram of mango exocarp tissues were ground with liquid nitrogen, then was homogenized with 10 mL methanol 80% (v/v). After centrifugation at 4 °C and 12,000× *g* for 20 min, the supernatant was collected for analysis of total phenolics and flavonoids contents. Total phenolics, flavonoids and lignin contents were measured according to our previous method [16]. Total phenolics and flavonoids content was expressed as mg g^−1^ fresh weight (FW). Lignin content was expressed as A280 kg^−1^. 

### 2.4. Measurement of SA and JA Contents 

Exocarp samples (0.5 g) were used for JA and SA extraction according to the method of Zhu et al. [19]. Analysis of JA and SA contents were conducted using Ultra Performance Liquid Chromatography (UPLC). The obtained sample (10 μL) was injected into UPLC (model: Waters UPLC ACQUITY 1-Class, Waters Corp., Milford, MA, USA) equipped with ACQUITY UPLC BEH C18 Column (1.7 µm, 1.0 × 50 mm). An optimized gradient of mobile phases of 65% A (Methanol) and 35% B (0.1% formic acid in water) for JA, and 60% A and 40% B for SA was used with a flow rate of 0.8 mL/min. Detecting wavelengths was 230 nm and 306 nm for JA and SA, respectively. JA and SA standards (Sigma-Aldrich, Milwaukee, WI, USA) were used to make a standard curve. SA and JA content was expressed as ng g^−1^ FW. 

### 2.5. Analysis of Defense Related Enzyme Activities

Activities of phenylalanine ammonia lyase (PAL), β-1,3-glucanase (GLU) and chitinase (CHI) were measured using 3 g of mango exocarp tissue according to our previous methods [10]. Activities of PAL, GLU and CHI were respectively expressed as Unit (U) mg protein^−1^.

### 2.6. Protein Extraction and iTRAQ Analysis

Samples taken from 0 h, 18 h, and 4 d were used for proteomic analysis as explained below. Ten grams of exocarp tissues were collected for protein extraction according to our previous research [20]. Three independent biological replications were conducted for protein extraction and further analysis. BCA protein quantitative kit (TRANS, Beijing, China) was used for protein content analysis. Protein digestion was conducted with 5 mM dithiothreitol for 30 min at 56 °C and alkylated with 11 mM iodoacetamide for 15 min at room temperature in darkness. The protein sample was then diluted by adding 100 mM TEAB to urea concentration less than 2M. Finally, trypsin was added at 1:50 trypsin-to-protein mass ratio for the first digestion overnight and 1:100 trypsin-to-protein mass ratio for a second 4 h-digestion. After trypsin digestion, peptide was desalted by Strata X C18 SPE column (Phenomenex, Los Angeles, CA, USA) and vacuum-dried. Peptide was reconstituted in 0.5 M TEAB and processed according to the manufacturer’s protocol for iTRAQ kit. Briefly, one unit of iTRAQ reagent were thawed and reconstituted in acetonitrile. The peptide mixtures were then incubated for 2 h at room temperature and pooled, desalted, and dried by vacuum centrifugation. The tryptic peptides were fractionated into fractions by high pH reverse-phase HPLC using Agilent 300Extend C18 column (5 μm particles, 4.6 mm ID, 250 mm length). Briefly, peptides were first separated with a gradient of 8% to 32% acetonitrile (pH 9.0) over 60 min into 60 fractions. Then, the peptides were combined into 18 fractions and dried by vacuum centrifugation for further analysis. After fraction, the tryptic peptides were dissolved in 0.1% formic acid (solvent A), directly loaded onto a home-made reversed-phase analytical column (15-cm length, 75 μm i.d.). The gradient was comprised of an increase from 6% to 23% solvent B (0.1% formic acid in 98% acetonitrile) over 26 min, 23% to 35% in 8 min and climbing to 80% in 3 min then holding at 80% for the last 3 min, all at a constant flow rate of 400 nL min^−1^ on an EASY-nLC 1000 UPLC system (Thermo, Shanghai, China).

The peptides were subjected to NSI source followed by tandem mass spectrometry (MS/MS) in Q ExactiveTM Plus (Thermo, Shanghai, China) coupled online to the UPLC. The electrospray voltage applied was 2.0 kV. The m/z scan range was 350 to 1800 for full scan, and intact peptides were detected in the Orbitrap at a resolution of 70,000. Peptides were then selected for MS/MS using NCE setting as 28 and the fragments were detected in the Orbitrap at a resolution of 17,500. A data-dependent procedure that alternated between one MS scan followed by 20 MS/MS scans with 15.0 s dynamic exclusion. Automatic gain control (AGC) was set at 5E4. Fixed first mass was set as 100 m/z.

### 2.7. Protein Identification and Quantification

The obtained MS/MS data were processed using MaxQuant search engine (v.1.5.2.8). Tandem mass spectra were searched against *Mangifera indica* database (52,097 sequences) concatenated with reverse decoy database. Trypsin/P was used as cleavage enzyme allowing up to 2 missing cleavages. The mass tolerance for precursor ions was set as 20 ppm in First search and 5 ppm in Main search, and the mass tolerance for fragment ions was set as 0.02 Da. Carbamidomethyl on Cys was specified as fixed modification and oxidation on Met was specified as variable modifications. FDR was adjusted to <1% and minimum score for peptides was set >40. Differentially expressed proteins (DEPs) were identified as those with normalized total intensity ratios >1.3 or <0.77 combined with *p*-value < 0.05.

### 2.8. Informatics Analysis of Differentially Accumulated Proteins

Gene Ontology (GO) annotation was derived from the UniProt-GOA database (http://www.ebi.ac.uk/GOA/). Then WEGO (Web Gene Ontology Annotation Plot) [21] was used to visualize plotting GO annotation results based on biological process.

Protein–protein interaction (PPI) analysis of differentially accumulated proteins was conducted according to our previous research [22]. After searched against *Arabidopsis thaliana* database lodged in the STRING database (version 11.0; http://string-db.org), the matching proteins were used for PPI analysis with a confidence score of 0.400. Then, the PPI network was constructed and displayed using Cytoscape (version 3.7.0) software.

The differentially regulated proteins were further graphically analyzed and visualized using MapMan (3.6.0RC1). Firstly, proteins were aligned against the *Arabidopsis thaliana* proteome TAIR9 (www.arabidopsis.org) using blastx with an E-value < e–5 and identity >60%. Then, Arabidopsis MapMan pathways of Response and Biotic stresses were adopted.

### 2.9. RNA Extraction and Real Time-Quantitative PCR (RT-qPCR) Analysis

RNA extraction was conducted according to our previous approach [23]. After digestion with DNase I (TIANGEN, Beijing, China), the DNA-free RNA was used to synthesize the first strand of cDNA using the PrimeScriptTM RT reagent Kit (Takara, Dalian, China) according to the manufacturer’s instructions. A total of 20 genes encoding some important differently accumulated proteins were selected for real-time quantitative PCR (RT-qPCR). RT-qPCR was performed according to our previous method [23] using the primers which were shown in Table S1. Three biological replicates were conducted.

### 2.10. Statistical Analysis

All the experiments were performed randomly with three biological replicates. Data were expressed as the mean ± standard errors. The statistical analysis was conducted using SPSS version 7.5 (SPSS Inc., Chicago, IL, USA).

## 3. Results

### 3.1. Establishment of BABA-Induced Priming Defense Against C. gloeosporioides in Mango Fruit

**Figure 1:**
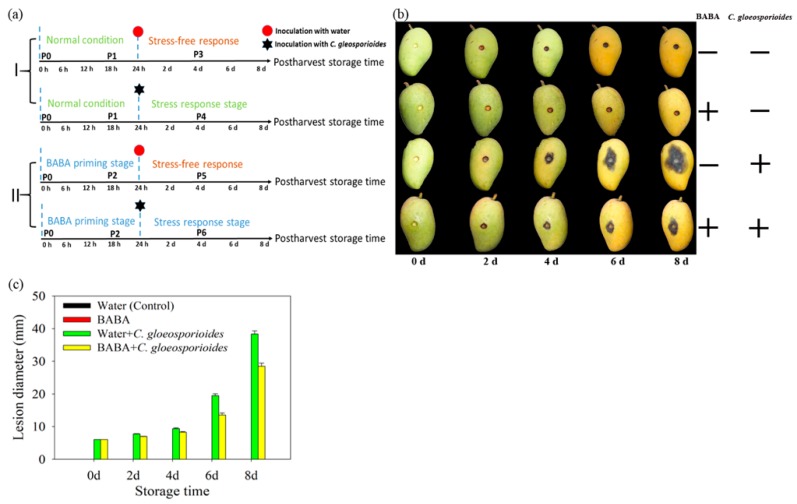
Outline of the platform used for β-aminobutyric acid (BABA)-priming mechanism investigation. (**a**) Mango fruit were non-primed (**I**) or BABA-primed (**II**) before inoculation of *C. gloeosporioides* during postharvest storage; (**b**) Visual image of anthracnose disease in mango fruit after different treatments; (**c**) Lesion diameter of mango fruit after different treatments.

In this study, we established a BABA-induced priming platform in mango fruit to elucidate clearly the regulating mechanism of BABA-induced priming against *C. gloeosporioides* (Figure 1a). As shown in Figure 1b, anthracnose gradually became severe in control fruit after *C. gloeosporioides* inoculation during storage at 25 °C. BABA treatment effectively inhibited the lesion expansion of anthracnose caused by *C. gloeosporioides*, indicated by smaller lesion diameter in BABA + *C. gloeosporioides* (Figure 1c). In all, set II fruits (primed by BABA) performed much better than Set I fruits (non-primed fruits) (Figure 1b,c).

### 3.2. Effect of BABA Treatment on Defense-Related Metabolites Contents in Mango Fruit

**Figure 2:**
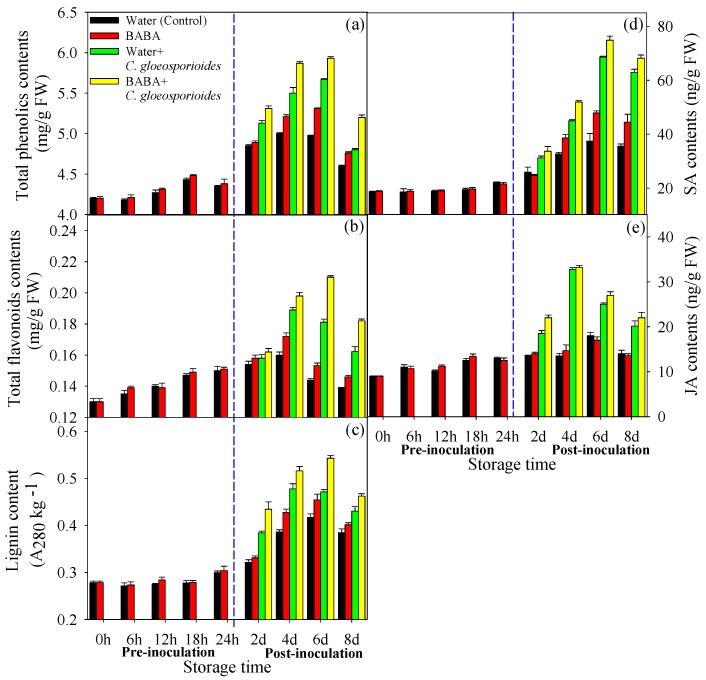
Effect of BABA treatment on total phenolics (**a**), total flavonoids (**b**), lignin content (**c**), SA (**d**), and JA (**e**) content in mango fruit. Data presented are means ± standard errors (n = 3).

Total phenolics, flavonoids and lignin contents initially increased and peaked at day 6 in both control and BABA-treated fruits during storage. Compared with control, BABA treatment had no influence on these two metabolites before inoculation of *C. gloeosporioides*. However, from day 4 to day 8 of storage, total phenolics, flavonoids and lignin contents in BABA-treated fruit were significantly higher than those in control fruits (Figure 2a–c). As shown in Figure 2a–c, inoculation of *C. gloeosporioides* accelerated the increase of these three metabolites in both control and BABA-treated fruits. Furthermore, total phenolics, flavonoids and lignin contents in BABA + *C. gloeosporioides* were higher than those in Water + *C. gloeosporioides* during most of storage, except flavonoids content at day 2 (Figure 2a–c).

SA and JA are involved in plant defense against pathogen. In this study, changing of SA content showed similar mode with that for lignin (Figure 2d). BABA treatment induced the SA accumulation from day 4 to day 8 compared with control, SA content in BABA + *C. gloeosporioides* was also higher than those in Water +*C. gloeosporioides* from day 4 to day 8 (Figure 2d). With respect to JA, its content increased slightly during storage and BABA treatment had no influence on JA content (Figure 2e). Although inoculation of *C. gloeosporioides* induced the increase of JA content, there were no significant difference between BABA + *C. gloeosporioides* and Water + *C. gloeosporioides*, except day 2 (Figure 2e).

### 3.3. BABA Treatment Enhanced Defense-Related Enzyme Activities in Mango Fruit

**Figure 3:**
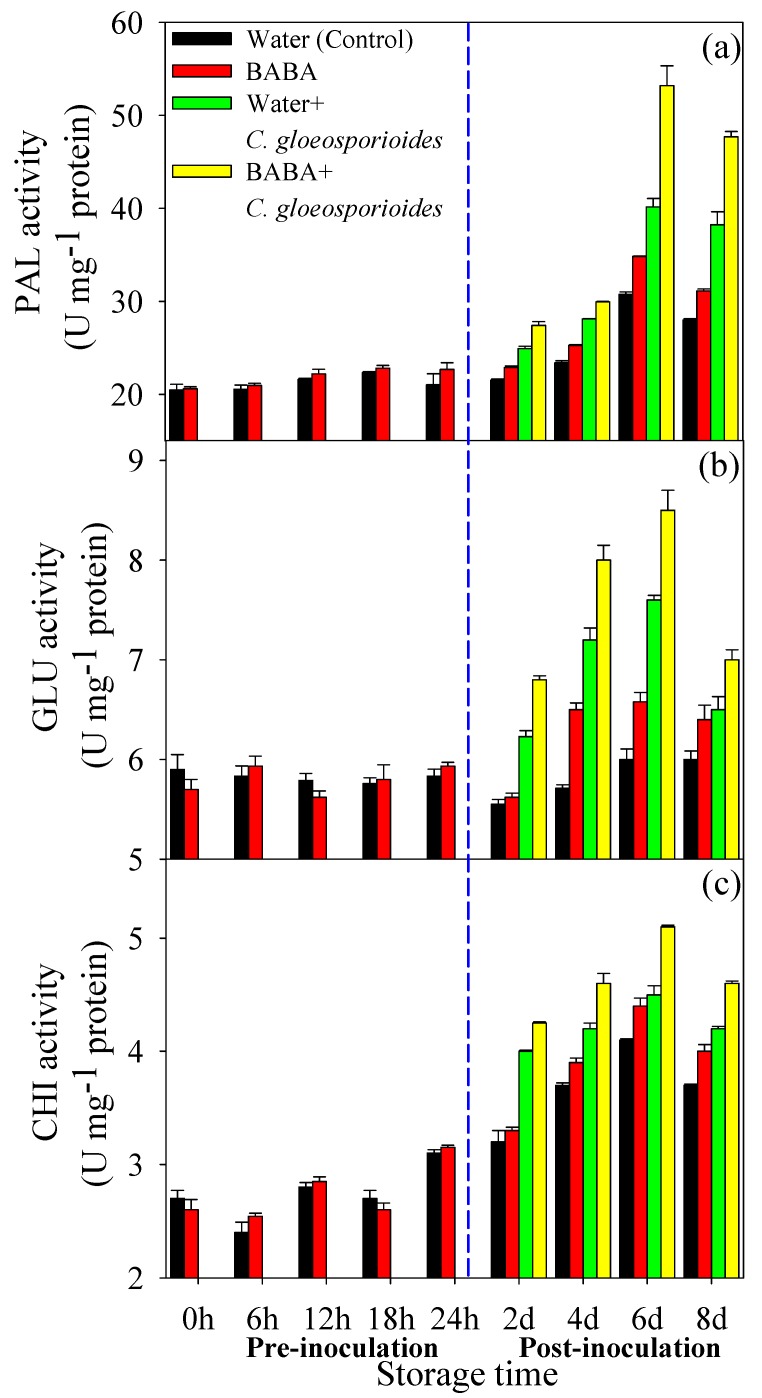
Effect of BABA treatment on β-1,3-glucanase (GLU) (**a**), phenylalanine ammonia lyase (PAL) (**b**) and chitinase (CHI) (**c**) activities in mango fruit. Data presented are means ± standard errors (n = 3).

PAL activity increased within the first 6 days of storage, then declined slightly (Figure 3a). During early storage time (first 24 h), BABA treatment had no effect on PAL activity, but from day 2, BABA induced higher PAL activity than control (Figure 3a). In response to infection of *C. gloeosporioides*, fruits treated with BABA possessed the highest PAL activity among fruit with these four treatments (Figure 3a). For GLU and CHI, BABA treatment did not affect their activities within the first 2 days of storage (Figure 3b,c). From day 4 to day 8, BABA-treated fruits had higher GLU and CHI activities. Similar with changing of PAL activity, GLU and CHI activities in BABA + *C. gloeosporioides* were higher than those in Water + *C. gloeosporioides* from day 2 to day 8 (Figure 3b,c).

### 3.4. Proteomic Changes During Different Phase of Pathogen-Stress Priming

To gain insight into the molecular changes associated with BABA-triggered defense against *C. gloeosporioides* infection, we focused on changes in the proteome during whole priming stages in mango exocarp. We collected samples at different time-points before priming (0 h), during priming (18 h), and exposure to pathogen (4 d). In this study, we identified 6009 proteins, among which 4860 proteins have been quantified. We chose the 18 h time point after treatments with BABA and water, which represented a time point of priming stage without *C. gloeosporioides* stress. Further, to address the events in the post-challenge primed stage, we chose the 4-d time point of post-inoculation as a representative, because the most vigorous physiological metabolism (e.g., drastic respiratory climacteric) could occur at this period of postharvest mango fruit.

### 3.5. Identification of Differentially Accumulated Proteins Associated with Early BABA-Induced Priming in Mango Fruit

**Figure 4:**
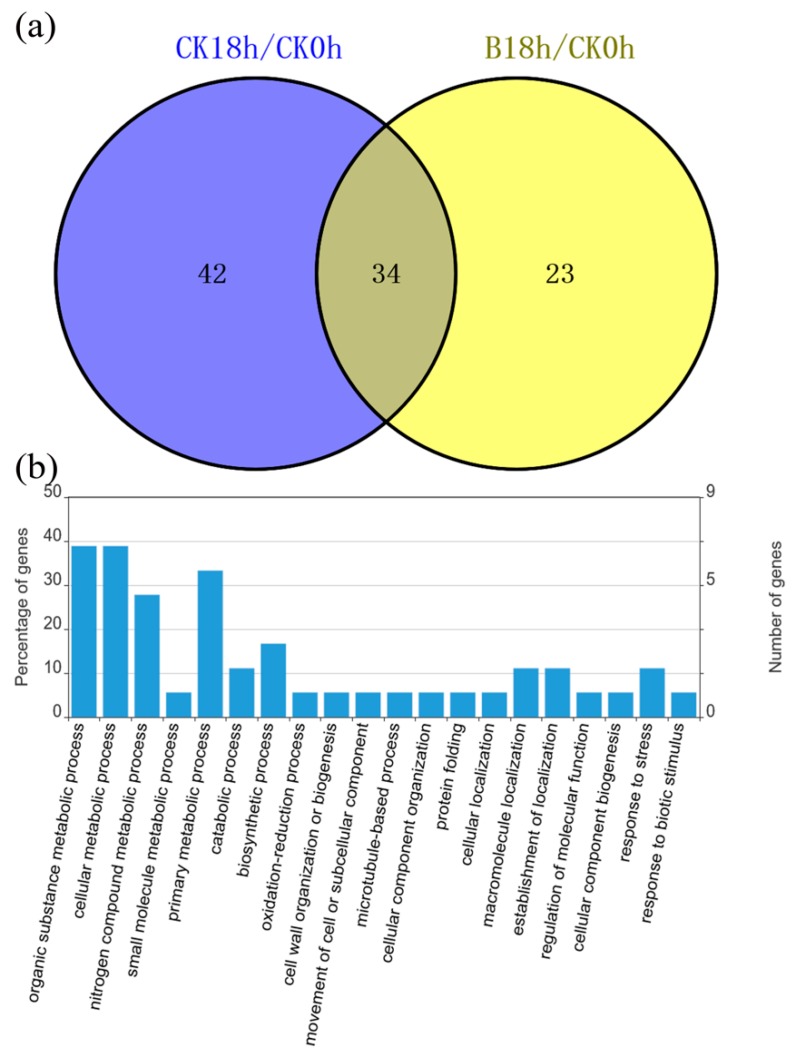
Differentially regulated proteins at priming stage. (**a**) The number of proteins regulated by priming at 18 h; (**b**) Gene Ontology (GO) analysis of the proteins differentially regulated at priming stage.

To identify proteins that might orchestrate priming, we compared the control samples with BABA-treated fruit. Upon BABA treatment, 57 proteins were differentially regulated. To exclude the ripening or other physiological influence, we also compared the difference between CK18h and CK0h. We identified 23 proteins that were only regulated by BABA priming, including 5 up-regulated and 18 down-regulated (Figure 4a, Table S2). In addition, for the 34 proteins that were both regulated in B18h and CK18h compared to 0 h, there were 5 down-regulated proteins in BABA-primed fruit relative to non-primed fruit (Table S2). According to GO analysis, these differentially expressed proteins (DEPs) were mainly involved in metabolic processes (primary metabolic, organic substance metabolic, nitrogen compound metabolic, etc.), responses to stress, establishment of localization and so on (Figure 4b). Among these proteins, non-specific lipid-transfer protein 1 (TRINITY_DN99185_c0_g1_m.64726) was significantly induced by BABA treatment (Table S2). Two UDP-glycosyltransferase (TRINITY_DN90262_c1_g2_m.59684 and TRINITY_DN90262_c1_g5_m.59687), callose synthase 10 (TRINITY_DN89300_c2_g1_m.56455), translational activator GCN1 (TRINITY_DN89756_c0_g10_m.57865), translation initiation factor 3 subunit A (TRINITY_DN90321_c1_g3_m.59938) and mitogen-activated protein kinase 1 (TRINITY_DN81561_c2_g3_m.41820) were also specifically down-regulated in response to BABA treatment (Table S2). The accumulation of chitinase CHI1 (TRINITY_DN17497_c0_g1_m.12170), pathogenesis-related protein STH-2-like (TRINITY_DN72717_c1_g1_m.34085), miraculin (TRINITY_DN65762_c0_g1_m.30030) and chalcone synthase 1-like (TRINITY_DN76531_c0_g1_m.36902) in BABA fruit were all lower than that in Water fruit (Table S2). In all, before inoculation of pathogen, most of the proteins in mango exocarp showed decreased patters in response to BABA priming.

### 3.6. Differentially Accumulated Proteins in BABA-Primed and Non-primed Fruit in Response to C. gloeosporioides Inoculation

**Figure 5:**
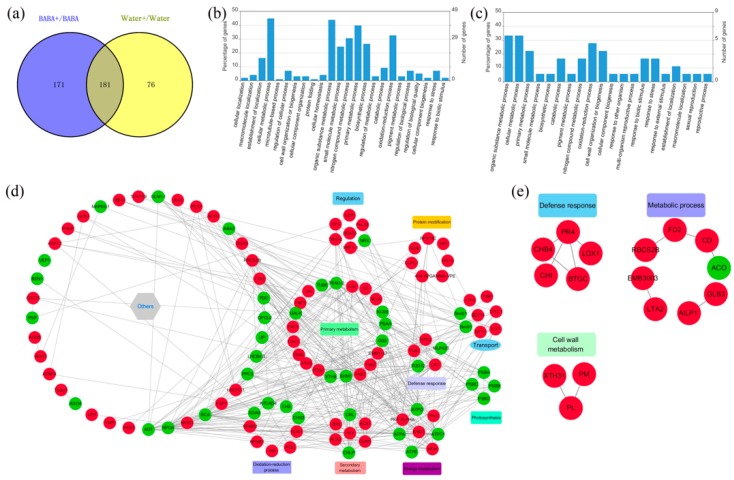
Differentially expressed proteins in response to *C. gloeosporioides* infection in primed fruit and non-primed plants. (**a**) Venn diagrams showing the number of differentially regulated proteins. BABA+ represents BABA + *C. gloeosporioides* fruits while Water+ represents Water + *C. gloeosporioides* fruits. (**b**) GO analysis of the proteins only differentially regulated in BABA primed fruit. (**c**) GO analysis of the proteins differentially expressed between BABA+ *C. gloeosporioides* and Water + *C. gloeosporioides*. (**d**) Protein–protein interaction (PPI) analysis of proteins only differentially regulated in BABA primed fruit. (**e**) PPI analysis of proteins differentially expressed between BABA + *C. gloeosporioides* and Water + *C. gloeosporioides*. Red color in D and E represented upregulation while green color represented downregulation.

Then, we investigated why BABA-primed fruit exhibited stronger resistance against *C. gloeosporioides* infection. At day 4, BABA treatment showed weak influence on protein accumulation without *C. gloeosporioides* challenge with only 13 differentially regulated proteins (Figure S1). However, with inoculation of *C. gloeosporioides*, the differentially regulated proteins between BABA-primed and non-primed fruit were significant. First, we analyzed the different responses of BABA-primed and non-primed fruit to *C. gloeosporioides* inoculation. Our data showed that more differentially regulated proteins were identified following infection with *C. gloeosporioides* in BABA-primed fruits than non-priming fruit. Among these proteins, 171 proteins were specifically differentially regulated in BABA-primed fruit in response to *C. gloeosporioides* infection including 106 up-regulated and 65 down-regulated proteins (Figure 5a, Table S3). GO analysis showed that these proteins were mainly involved in metabolic process (primary metabolic, secondary metabolic, etc.), oxidation-reduction process, response to stress and so on (Figure 5b). Next, we found 181 proteins that were differentially regulated in both BABA primed and non-primed fruit in response to pathogen (Figure 5a). However, there were only 40 up-regulated proteins and 2 down-regulated proteins in BABA + *C. gloeosporioides* compared to Water + *C. gloeosporioides* (Table S3). Our data showed that, besides metabolic process and oxidation-reduction process, response to biotic stimulus was also the main biological process that these differentially regulated proteins involved in (Figure 5c). Altogether, the largest group of differentially regulated proteins were found in BABA + *C. gloeosporioides*. This list of proteins included those that were responsible for higher resistance against *C. gloeosporioides* infection. It should be noted that major latex protein (MLP)-like protein 28 (TRINITY_DN73690_c2_g2_m.34754), pathogenesis-related protein PR-4 (TRINITY_DN59793_c0_g1_m.27015) and pathogenesis-related protein STH-2-like (TRINITY_DN72717_c1_g1_m.34085) were up-regulated in BABA-primed fruit after *C. gloeosporioides* infection (Table S3). This would explain why the primed fruits tolerated exposure to pathogen while the non-primed fruits did not.

PPI analysis was conducted to further investigate the interaction of the differentially accumulated proteins after BABA treatment. For proteins that were specifically differentially regulated in BABA-primed fruit in response to *C. gloeosporioides*, PPI analysis indicated that, several different functional categories with a total of 112 nodes (Supplementary Table S3) (Figure 5d). For the proteins that were up-regulated in BABA + *C. gloeosporioides* compared to Water + *C. gloeosporioides,* they were classified into defense response, cell wall metabolism and metabolic process with a total of 16 nodes (Figure 5e). Altogether, PPI analysis indicated that the differentially regulated proteins involved in diverse biological processes might interact and act synergistically in BABA-priming defense.

**Figure 6:**
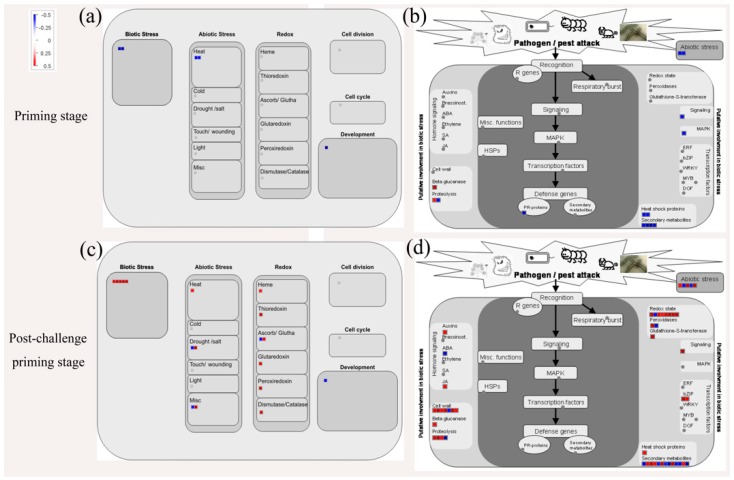
Proteins that were differentially regulated in BABA-primed fruit at priming stage and post-challenge priming stage were graphically analyzed and visualized using the MapMan. Proteins that were differentially regulated in BABA-primed fruit at priming stage involved in cell defense (**a**) and biotic stress (**b**); proteins that were differentially regulated in BABA-primed fruit at post-challenge priming stage involved in cell defense (**c**) and biotic stress (**d**). Up-regulated proteins were presented in red while down-regulated proteins in blue.

We also conducted MapMan analysis to perform metabolic clustering analysis of differentially regulated proteins that are responsible for cell defense and biotic stress. At priming stage, few metabolic processes were affected by BABA treatment (Figure 6a,b) while much more proteins involved in cell defense and biotic stress were identified in BABA-primed fruit at post-challenge priming stage (Figure 6c,d).

### 3.7. Gene Expression Analysis

**Figure 7:**
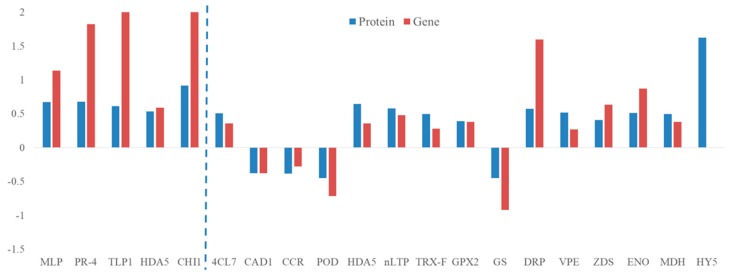
Effect of BABA treatment on expression level of selected genes in mango fruit.

In order to evaluate the transcript changes of differentially accumulated proteins, we conducted RT-qPCR. As shown in Figure 7, all the gene expression were consistent with protein level, except HY5.

Additionally, we also conducted gene expression analysis of NPR1 and TGA genes that are involved in SA signaling. As shown in Figure S2, during the whole storage, NPR1 gene expression increased at first 6 days storage, then decreased. Before inoculation, BABA treatment had no significant influence on the NPR1 expression at first 12 h. However, at 18 and 24 h, BABA-treated fruit had higher expression level than control fruit. Inoculation of pathogen significantly induced the NPR1 gene and fruit treated with BABA had much higher expression level than that in Water (Control) fruit. Similarly, BABA slightly changed the expression level of TGA before inoculation of pathogen. *C. gloeosporioides* inoculation induced the increase of TGA expression. Again, BABA-treated fruit had higher expression level of TGA than control in response to *C. gloeosporioides* infection (Figure S2).

## 4. Discussion

Priming is a state that can be induced by appropriate stimuli whereby plant exhibit a rapid and strong activation of defense mechanism upon exposure to subsequent stresses. For now, some achievements were made on mechanisms of priming [4], however, molecular understanding of priming still remained elusive. Hence, identification of more co-activators or signaling proteins in the priming defense process would be in favor of determining the underlying mechanisms involved in BABA mediated priming. In this study, we established a comprehensive BABA-induced priming platform to gain an insight into the mechanism of BABA-induced resistance against *C. gloeosporioides* by priming. The proteomic approach were employed in the present study to shed light on the dynamics of protein abundance which provided an improved picture about the resistance mechanism of BABA primed host against pathogen.

### 4.1. Identification of Protein Accumulation During Early Priming Stage

Till now, the research on the BABA priming defense mainly focused on the defense response of host after pathogen infection. The priming phase is also important preparing stage for the establishment of post-challenge primed state. The priming state can be triggered by multifarious priming stimuli, by which the levels of various primary and secondary metabolites, enzymes, hormones, and other molecules are slightly altered, putting the plant in a standby and/or alarmed state [3]. However, there is still lacking in the details for priming stage (the preparation or acquisition of priming defense). In addition, the connection of priming stage and post-challenge primed stage also need further investigation. Hence, one of the focuses in the current study was on those proteins that were uniquely regulated in primed fruit but not in non-primed mango fruit in both priming and post-challenge primed stages. This list of proteins should include those that might control the acquisition and/or maintenance of priming, thereby allowing primed fruits to survive subsequent exposure to stress. 

Non-specific lipid-transfer proteins (nsLTPs) are cysteine-rich lipid binding proteins that have been reported to mediate plant defense against pathogens and other environmental stresses through promoting the formation of hydrophobic protective layers [24,25]. Blein et al. [24] also suggest that several LTPs might possess direct in vitro antimicrobial properties by increasing membrane permeability of microbes. Salcedo et al. [26] showed that some nsLTPs in *A. thaliana* could be involved in plant lipid signal transduction, promoting the long-distance signaling during systemic acquired resistance. A previous research found that several nsLTP isoforms of *Trichoderma harzianum* T39-treated grapevines increased upon *Plasamopara viticola* inoculation, indicating specific involvement of LTP isoforms in defense responses of plant [27]. Our proteomic results showed that one nsLTP 1 (TRINITY_DN99185_c0_g1_m.64726) was up-regulated in the BABA-treated mango fruit in early priming stage, contributing to the preparation of the defense response or signaling in mango fruit. Importantly, this protein was further up-regulated in BABA-primed fruit following inoculation with *C. gloeosporioides*. A recent research reported that some primed-induced genes constitute the memory genes through retaining their transcriptional level after prime treatment to increase plant tolerance to subsequent exposure stress [28]. Our results suggest that nsLTP 1 (TRINITY_DN99185_c0_g1_m.64726) might be responsible for the acquisition and maintenance of priming, contributing to stronger resistance against *C. gloeosporioides* infection in BABA-treated mango fruit.

Pathogenesis-related proteins and chitinase are well known for their vital roles in plant defense against biotic stress [29]. Compared to control fruit, the accumulation levels of chitinase CHI1 precursor (TRINITY_DN17497_c0_g1_m.12170) and pathogenesis-related protein STH-2-like (TRINITY_DN72717_c1_g1_m.34085) were lower in BABA-treated fruit during priming stage. However, after infection of *C. gloeosporioides,* both proteins were rapidly accumulated in Water + *C. gloeosporioides*-treated and BABA + *C. gloeosporioides*-treated fruit, but the accumulation amount of latter was higher than that of former. These results suggested a stronger activation of defense responses in primed mango fruit when subsequently challenged by fungi infection. During the priming stage, slight alteration (though down-regulated) of the accumulation level of chitinase CHI1 and pathogenesis-related protein STH-2 might put the fruit in a standby state without significant fitness costs. However, these proteins in mango fruit were rapidly induced once fruit combating the pathogen infection. Altogether, we postulated that these defense-related proteins might be related to the establishment of the primed state in mango fruit.

Many defense compounds of plants are stored in a non-active glucosylated form and can be activated via hydrolysis of the glucosidic linkage catalyzed by beta-glucosidases upon stress [30]. Glucan endo-1, 3-beta-glucosidase (beta-1, 3-glucosidase) was reported to be implicated in the defense of plants against pathogens [31]. In addition, glucan endo-1, 3-beta-glucosidase was also reported to be related to BABA-induced tolerance against abiotic stress in barley [15]. In this study, compared with control fruit, glucan endo-1, 3-beta-glucosidase 7 (TRINITY_DN64071_c0_g2_m.29098) was induced in BABA fruit at 18 h of storage. The higher amount of the glucan endo-1, 3-beta-glucosidase at early priming stage can prepare fruit for hydrolysis of defense compounds upon pathogen attack. In contrast, the UDP-glycosyltransferase (UGT) was reported to play an essential role in the glycosylation of some defense compounds, such as isoflavonoids [32]. Our results showed that UDP-glycosyltransferase (TRINITY_DN90262_c1_g2_m.59684 and TRINITY_DN90262_c1_g5_m.59687) were down-regulated in BABA-exposed fruit after 18 h of storage while not identified in control fruit, also indicating lower glucosylation of defense compounds in BABA fruit. Whereas, all these proteins were not differentially regulated in BABA-primed fruit after *C. gloeosporioides* infection. This phenomenon was consistent with one of the characteristics of the priming stage: Starting after perception of the priming stimulus and extinction with the exposure to a challenge. We reasonably put forward the hypothesis that glucan endo-1, 3-beta-glucosidase and UDP-glycosyltransferase might be just responsible for the acquisition of priming by BABA, not for maintenance of priming.

Altogether, the present study established a platform aiming to unveil the essence of acquisition and/or maintenance of BABA-induced defense response against pathogens by priming at the protein scale.

### 4.2. BABA-Primed Defense Against C. gloeosporioides Infection in Mango Fruit

Previous research indicated that the physiological [5], biochemical [33] and molecular changes [1] at the cellular level in BABA-treated plants occurred only upon infection with pathogens, suggesting that BABA-induced resistance could be involved in priming of defense response. Our previous research also confirmed the BABA-induced resistance by priming in response to *C. gloeosporioides* infection in mango fruit [10]. In the present study, BABA-primed fruit showed higher disease resistance as indicated by restricted expansion of anthracnose lesion (Figure 1). Before inoculation of *C. gloeosporioides,* BABA-treated fruit showed no changes in defense-related metabolites content and enzymes activity. Differently, subsequent exposure to *C. gloeosporioides* stimulated more accumulation of defense metabolites in BABA-primed fruit than that in non-primed fruit (Figure 2 and Figure 3). Previous research reported that low concentration of BABA, such as 10 mM, induced a defense through priming mode, whereas higher concentrations of BABA (100 or 500 mM) activated a direct defense in strawberries [5]. Here, we also compared with the differentially proteins in Water and BABA-treated fruit at 4 d without *C. gloeosporioides* inoculation. Proteomic results showed that, BABA treatment had no significant influence on the protein accumulation compared to Control fruit after 4 d storage (Figure S1). Especially, no defense-related proteins were identified. Additionally, in our previous research, we have demonstrated that BABA treatment decreased natural disease incidence but not affect the softening, ethylene production, respiration rate, total soluble solids content and total titratable acidity in mango fruit during storage at 25 °C, ruling out the possibility of interference by fruit ripening [10]. Hence, we proposed that induction of defense response by 50 mM BABA could involve in mediation of priming. In addition, MapMan results showed that much more proteins involved in cell defense and biotic stress were identified in BABA-treated fruit at post-challenge primed stage (Figure 6).

Secondary metabolites such as phenolics and flavonoids played important roles in response to infection of plant pathogenic fungi [34]. Moreover, these two metabolites were reported to contribute to UV-C induced protection of strawberry leaves against *Mycosphaerella fragariae* by priming [34]. Consistently, the present study confirmed that BABA application could markedly increase phenolics and flavonoids contents. However, the regulating mechanism of BABA treatment on these defense related metabolites biosynthesis is still poorly understood. In the current study, transcription factor HY5-like (bZIP) (TRINITY_DN69400_c0_g1_m.32045) was only up-regulated in BABA-primed fruit in response to *C. gloeosporioides* infection. Previous research reported that HY5 upregulated the R2R3MYB transcription factor gene MYB12 that is considered a key activator of flavonol biosynthesis [35]. Additionally, disruption of hy5 in mutants of Arabidopsis showed lower flavonoid production and more susceptibility to UVB stress-induced injury [36]. It is therefore reasonable to deduce that HY5 was likely a key factor in BABA induced higher accumulation of flavonoids in response to infection of *C. gloeosporioides*. Importantly, MapMan analysis indicted that bZIP transcription factors were up-regulated and involved in biotic stress response in BABA-primed fruit at post-challenge priming stage (Figure 6d). Interestingly, gene expression level of *HY5* did not change in BABA-primed fruit in response to *C. gloeosporioides* infection (Figure 7). We postulated that the difference of *HY5* expression level might appear earlier than the protein level of HY5 due to its prominent role in transcriptional regulation.

Flavonoids and lignin have been shown to promote the plant response to a variety of biotic and abiotic stresses [16,37]. Flavonoid and lignin compounds can be synthesized through the phenylpropanoid pathway [32]. Here, BABA+ *C. gloeosporioides* fruit had the highest PAL activity compared to other groups (Figure 3), indicating that enhancement of phenylpropanoid pathway could be involved in defense response by BABA-induced priming in mango fruit. In the phenylpropanoid pathway, 4-coumarate-CoA ligase (4CL) is also key enzyme and contributes to the biosynthesis of phenolics, flavonoids, and lignins [38]. The present study noted a remarkable upregulation in BABA-primed fruit upon pathogen inoculation. Accordingly, higher flavonoids and lignin contents were found in BABA+ *C. gloeosporioides* fruit compared to Water + *C. gloeosporioides* (Figure 2b,c), which was consistent with protein and enzyme activity level. Similarly, Xie et al. [37] reported that γ-aminobutyric acid, another non-protein amino acid with priming effect, could regulate the pathway of flavonoid biosynthesis in poplar under normal or stress conditions through influencing expression of key genes involved in flavonoid biosynthetic pathway. Hu et al. [16] also pointed that activation of PAL and 4CL activities contributed to NO-induced resistance of mango fruit to anthracnose. Thus, it could be postulated that the BABA-primed mango fruit might enhance phenylpropanoid metabolism to induce the higher accumulation of flavonoids and lignin in fruit, conferring protection against pathogen infection. In response to pathogen attack, plants can strengthen cell wall structure via regulating lignin biosynthesis. Therefore, higher lignin content also suggest that lignin-mediated defense barrier in mango fruit might play important role in BABA-induced priming defense against pathogen infection. 

Recent study suggested that accumulation of phenolics might be regulated partly by the SA hormones signaling [34]. SA signaling was also reported to be associated with priming defense [14,39]. Consistent with UVC-induced resistance by priming in strawberry leaf [34], BABA + *C. gloeosporioides* fruit showed higher SA content than Water+ *C. gloeosporioides* during post-challenge primed stage (Figure 2c), which was accompanied with synchronous change in phenolics content (Figure 2a). Furthermore, the related genes in SA signaling pathway, including NPR1 and TGA were also up-regulated by BABA treatment (Figure S2). In response to *C. gloeosporioides* infection, higher accumulation of SA in BABA-primed than that in control fruit can be viewed as an indication that the BABA-primed fruit were able to sense and respond to the pathogen faster. In our case, SA biosynthesis as well as related signals transduction processes were amplified in BABA-primed fruit, which might result in faster and higher accumulation of phenolics and PR proteins in response to pathogen infection. Consistent with our results, Zimmerli et al. [7] also reported that BABA treatment potentiated the SA-regulated defense mechanisms in Arabidopsis.

Besides, the present study also identified differentially accumulated proteins involved with biosynthesis of secondary metabolites with higher accumulation in BABA + *C. gloeosporioides* fruit, including peroxidase (TRINITY_DN115674_c0_g1_m.4242, TRINITY_DN72550_c0_g1_m.33984) and ZDS (TRINITY_DN67292_c0_g1_m.30843) and phytoene synthase (TRINITY_DN85387_c0_g1_m.47412). Phytoene synthase (PSY) catalyzes the condensation of geranylgeranyl pyrophosphate and ZDS (ζ-carotene desaturase) is responsible for the cyclization of lycopene. They are important enzymes involved in biosynthesis pathway of carotenoid [40,41]. Oxidoreductase, for example, PODs catalyze the oxidation of phenolic compounds and other substrates through consumption of H_2_O_2_, have been shown in correlation with the accumulation of secondary metabolites [42]. These proteins were reported to be involved in plant induced-defense responses by priming [40,42]. PPI analysis also suggested these secondary metabolism-related proteins had important roles in mango fruit in response to pathogen infection (Figure 5d). In all, BABA-treated fruit exhibited an increased capacity to regulate secondary metabolites only when the fruit were inoculated with *C. gloeosporioides*, which was confirmed by MapMan analysis (Figure 6d). 

### 4.3. Identification of Vital Pathways Involved in BABA-Primed Defense Response to Pathogen Infection

As described by previous research, priming stage was characterized as uneventful, in which the various enzymes were kept in a standby state, however, strong regulation of defense-related proteins occurred only in the post-challenge primed phase [3]. In addition, the primed state could be based on the modification of one or more signaling proteins that still remain inactive [43]. Therefore, stimulation of cellular inactive proteins was reported to play an important role in cellular signal amplification of priming defense and post-transcriptional regulation, for example, phosphorylation modification plays an essential role in this process [4].

Calcium-dependent protein kinases (CDPKs) are widely and specifically expressed in plants and were reported to be involved in plant response to different stresses [44]. Importantly, CPK21 was reported to contribute to abiotic stress signaling in Arabidopsis [45], rice [44] and possibly via post transcriptional regulation of transcription factors [44]. Zhao et al. reported that CDPK gene was involved in calcium signal transduction and activation of downstream calcium-binding transcription factors, such as NAC and MYB in nanometer CaCO_3_-treated herbaceous peony [46]. Here, the specific upregulation of CDPK (TRINITY_DN70734_c0_g1_m.32913) in BABA + *C. gloeosporioides* fruit might lead to the initiation of faster downstream signaling, which was beneficial for more rapid and robust fight against pathogen than control fruit. Certainly, the exact target defense genes or downstream signaling pathways of CDPK warrant further research. Diacylglycerol kinase (DAGK)-mediated signaling is one important component of lipid signaling pathway and plays important roles in plant growth and development, stress and hormone response, and disease resistance [47]. In this study, diacylglycerol kinase 5 (TRINITY_DN47829_c0_g1_m.22049) were only up-regulated in BABA + *C. gloeosporioides* fruit. Similarly, in BTH-primed rice seedlings, the expression of diacylglycerol kinase gene (*OsBIDK1*) was higher than control seedlings after inoculation with *Magnaporthe grisea* [48]. Furthermore, overexpression of *OsBIDK1* in transgenic tobacco plants increased resistance against infection by tobacco mosaic virus and *Phytophthora parasitica* var [48]. Altogether, our result suggested that diacylglycerol kinase might also contribute to the enhanced resistance of mango fruit against infection by *C. gloeosporioides*.

MLP proteins belong to the Bet v 1 family and the participation of MLPs in biotic and abiotic stresses defense was reported in different higher plants [49,50]. Moreover, MLP28 in cotton acts as a positive regulator of ERF6 and contribute substantially to protection against *Verticillium dahliae* infection in cotton plants [50]. In the present study, higher accumulation of MLP-like protein 28 (TRINITY_DN73690_c2_g2_m.34754) was observed in BABA + *C. gloeosporioides* fruit (Table S3), which might contribute to strong defense against *C. gloeosporioides* infection. Importantly, our study identified a novel defense-related MLP protein in BABA-induced priming defense in mango fruit. Certainly, considering the role of MLP28 in enhancing transcriptional activation activity of some transcription factors [50], the identification of downstream target of MLP28 would be beneficial to elucidate the regulating mechanism of MLP28 in BABA-induced priming defense process.

A previous research reviewing the molecular aspects of priming emphasized the crucial role of chromatin modifications in priming defense in plants [4]. Histone deacetylation, catalyzed by histone deacetylase (HDAC), has been involved in response of plants to biotic and abiotic stresses [51]. Importantly, Yu et al. [51] indicated that silencing of SlHDA5 could result in decreased tolerance to salinity, drought and abscisic acid in tomato plant. In the present study, BABA-treated mango fruit exhibited an increased accumulation of histone deacetylase 5 isoform X1 (TRINITY_DN90601_c5_g11_m.61123) only when the fruits were inoculated with *C. gloeosporioides*. These findings suggested that chromatin modifications mediated by histone deacetylase appeared as an important strategy to induce faster and stronger disease resistance in priming defense by BABA in harvested mango fruit. The present PPI analysis also suggested that histone deacetylase might interact with other biological process to increase defense ability of mango fruit against pathogen attack (Figure 5d).

Plasma membrane proton pumps are involved in multiple physiological processes, including abiotic stress responses [52]. Proton pump-interactor 1 (PPI1), as one of the regulators of proton pumps, stimulates proton pump activity in vitro [52]. Moreover, PPI1 was reported to be involved in the adaptation responses of *Solanum tuberosum* L. to abiotic stress [52]. In this study, one proton pump-interactor 1-like (TRINITY_DN89014_c1_g1_m.55677) was identified with significant upregulation in BABA-treated mango fruit only when encountering pathogen challenge (Table S2). Till now, less information on the role of PPI1 in pathogen response was released. Our results first reported that BABA-induced resistance against pathogen might be related to the induction of proton pump-interactor 1 in postharvest mango fruit. Further understanding the mechanism of pathogen tolerance involving proton pump-interactor 1 will be investigated in future studies.

Interestingly, according to our proteomic results, for some defense-related proteins, besides their roles in establishment of the primed state, they were also differently accumulated in BABA-primed fruit during post-challenge stage. For example, three non-specific lipid-transfer proteins (TRINITY_DN83520_c2_g1_m.44432; TRINITY_DN99185_c0_g1_m.64726; TRINITY_DN107403_c0_g1_m.1949) were only up-regulated in BABA+ fruit (Table S3). Meanwhile, our results showed that the accumulation of pathogenesis-related protein PR-4 and chitinase were higher in BABA+ *C. gloeosporioides* compared to Water + *C. gloeosporioides* and they were involved in mango fruit defense response (Figure 5e). Considering the important roles of these proteins discussed above, our results further confirmed that, for some defense-related proteins, some were responsible for establishment of the primed state, but others contribute to higher resistance of mango fruit to *C. gloeosporioides*. Thaumatin-like protein is another defense-related protein, especially related to host resistance to pathogens [53]. Further, Ziogas et al. [54] found that thaumatin was BABA-responsive protein in citrus seeds in response to salinity stress. Here, thaumatin-like protein 1 (TRINITY_DN8225_c0_g2_m.42771) were higher in BABA+ *C. gloeosporioides* compared to that of Water + *C. gloeosporioides* (Table S3), suggesting that thaumatin-like protein might contribute to stronger resistance of BABA-primed fruit.

Another protein family with a well-established relationship to defense response is the antioxidant proteins/enzymes. Xu et al. [34] reported that coordinated action of antioxidant enzymes (catalase, CAT; glutathione reductase, GPX; ascorbate peroxidase, APX; etc.) in UV-C-treated strawberry leaves contributed to enhanced defense capacity against subsequent pathogen attack. Previous research also suggested that thioredoxin and glutaredoxin were involved the activation of defense response to downy mildew in *Trichoderma harzianum* T39- treated grapevine [27]. In the present study, after inoculation, upregulation of CAT (TRINITY_DN54558_c0_g1_m.24460), thioredoxin F-type (TRINITY_DN60144_c1_g1_m.27160), glutaredoxin (TRINITY_DN76625_c2_g1_m.37007) and glutathione peroxidase 2 (TRINITY_DN87504_c2_g4_m.51737, TRINITY_DN53600_c0_g2_m.24082) were only found in BABA+ (Table S3), indicating that the upregulation of these proteins could contribute to BABA-primed antioxidant defenses in response to pathogen infection. PPI results further suggested the involvement of CAT and GPX in defense response of mango fruit (Figure 5d). Pretreatments with BABA also improved citrus seed acclimation to salinity through altering the accumulation of redox-regulated enzymes, such as glutathione peroxidase [54]. MapMan results also indicated that thioredoxin and glutaredoxin were also involved in the activation of cell defense of mango fruit at post-challenge primed stage (Figure 6c). Consistent with our results, Wang et al. also confirmed that increased antioxidant ability contribute to the priming effects enabling the wheat to cope with drought stress [55].

Previous research revealed that L-glutamine could block BABA-mediated priming of the SA defense marker gene PR1 in Arabidopsis, leading to abolishment of BABA-induced resistance to both abiotic and biotic stresses [56]. In addition, L-glutamine might share a common transporter with BABA and might inhibit BABA translocation into the cell through competing for the transporter [56]. These findings indicate that action mode of both BABA and L-glutamine could be antagonized for each other in plants. In support of this idea, the present results showed that glutamine synthetase (TRINITY_DN6739_c0_g2_m.30908 and TRINITY_DN69464_c0_g1_m.32085) were down-regulated only in BABA-primed fruit in response to *C. gloeosporioides* infection. Taken together, glutamine synthesis inhibition might partially uncover the BABA-mediated priming defense mechanism in fruits. 

To date, the information on the physiological function of desiccation-related proteins (DRPs) is not known. Previous research found that DRPs are up-regulated when plants are infected by pathogens [57]. Zha et al. [58] identified a DRP isolated from bean, called MS-desi, which functioned as an antimicrobial agent in nectar by inhibiting glyoxylate cycle of microbes. In this study, desiccation-related protein PCC13-62 (TRINITY_DN71326_c0_g1_m.33275) was up-regulated in BABA+ *C. gloeosporioides* fruit, but not in Water + *C. gloeosporioides*. Together these results suggested that DRPs might be involved in BABA-induced priming defense against biotic stressors. 

Non-host resistance is the most common form of disease resistance in plants and is highly effective and durable with great agronomic importance [59]. Syntaxin-121 in *A. thaliana* was reported to provide the mechanistic link between non-host resistance and basal penetration resistance in monocotyledons and dicotyledons [60]. In addition, previous research indicated that BABA induced resistance against root-knot nematodes in rice was also involved with penetration resistance [61]. In this study, the syntaxin-121-like (TRINITY_DN74001_c0_g1_m.34921) was specifically up-regulated in BABA-treated fruit after *C. gloeosporioides* inoculation. Assaad et al. [59] found that syntaxin-121 appeared to have basal penetration resistance through regulating polarized secretion events that give rise to papilla formation to establish barriers in Arabidopsis to defense against *Blumeria graminis* f. sp. hordei (Bgh) fungus. According to these results, syntaxin-121 mediated penetration resistance might contribute to BABA-induced resistance by priming in mango fruit.

ABA-stress-ripening (ASR) proteins are small and basic proteins that are involved in fruit development and plant response to various abiotic stress, including water deficit, salt, cold and limited light [62]. However, the report on the role of ASR in biotic response was rare. In this study, abscisic stress-ripening protein (TRINITY_DN22594_c0_g1_m.13753) was down-regulated by BABA treatment after *C. gloeosporioides* infection. In addition, we did not note different accumulation of ASR in BABA and Control fruit without *C. gloeosporioides* inoculation. Therefore, we speculate the regulation of ASR by BABA might be more involved in priming defense response to fungal infection in postharvest mango fruit. Interestingly, previous research showed that ASR was involved in the regulation of cell wall metabolism, indicated by downregulation of SlPG and SlPME in SlASR1-RNAi tomato fruit [62]. Hence, we also postulate that downregulation of ASR in BABA+ *C. gloeosporioides* fruit might affect cell wall metabolism and reduce depolymerization and solubility of cell wall polysaccharides in mango fruit, thus contribute to improved mechanical strength of cell and enhanced defense capacity in BABA primed fruit. MapMan analysis also showed that cell wall metabolism played a significant role in BABA-primed resistance (Figure 6). The exact role of ASR in BABA priming warrant further investigation in our future research.

Proteolytic mechanisms have been associated with disease resistance in fruit [63]. Vacuolar processing enzymes (VPEs) are canonical cysteine proteases which are responsible for the maturation or activation of specific vacuolar proteins in plants [63]. Wang et al. [63] reported that VPE3 could contribute to resistance of tomato fruit against the fungal pathogen *Botrytis cinerea* via the cleavage of the serine protease inhibitor KTI4. Previous research also found that ATP-dependent Clp protease proteolytic subunit was induced in methyl jasmonate-primed *Zantedeschia aethiopica* in response to *Pectobacterium carotovorum* infection [42]. In the study presented here, one vacuolar-processing enzyme-like (TRINITY_DN82192_c1_g1_m.42683), one cysteine proteinase 15A (TRINITY_DN80053_c0_g3_m.40207) and ATP-dependent Clp protease ATP-binding subunit (TRINITY_DN90568_c9_g1_m.60994) were identified with significant upregulation in BABA-primed fruit in response to infection of *C. gloeosporioides*. Based on these results, we put forward the hypothesis that post-translational modification could also play important roles in BABA-primed defense against pathogen infection. Certainly, further studies focusing on potential targets of VPE in mango fruit will uncover the regulating mechanism of BABA-induced priming defense response in fruit.

Glycolysis, tricarboxylic acid (TCA) cycle and oxidative phosphorylation are important energy-generating pathways. ATP synthase (TRINITY_DN76636_c0_g1_m.37018, TRINITY_DN80063_c0_g1_m.40213, TRINITY_DN88262_c1_g3_m.53484 and TRINITY_DN85009_c2_g2_m.46734) were down-regulated in BABA-primed mango fruit in response to *C. gloeosporioides* inoculation (Figure 5d). In sum, when compared to Water + *C. gloeosporioides*, BABA + *C. gloeosporioides* fruit suffered less energy investments indicated by lower accumulation of proteins involved in ATP synthesis pathway. The observation made here was consistent with previous research, indicating priming defense induced by BABA resulted in the reduction of sugar costs for generating ATP in fruit, as demonstrated in strawberries [5]. Additionally, Vijayakumari et al. [64] pointed that excessive cost of metabolic energy in response to stress can strongly influence growth rate and fitness parameters in organisms. Considering the important role of energy in postharvest fruit storage [65], BABA-induced priming defense might involve lesser wastage of metabolic energy and make successful survival during the pathogen infection. Interestingly, our results also found that enolase (TRINITY_DN87366_c0_g1_m.51370), malate dehydrogenase (MDH, TRINITY_DN88557_c0_g3_m.54333), and transaldolase (TRINITY_DN9041_c0_g1_m.60333) which are involved in glycolysis and pentose-phosphate pathway, were up-regulated in BABA + *C. gloeosporioides* fruit (Figure 5d). We postulated that accelerated glycolysis might be favor for providing more backbone for other metabolic biosynthesis, such as secondary metabolism, but not for ATP synthesis.

## 5. Conclusions

**Figure 8:**
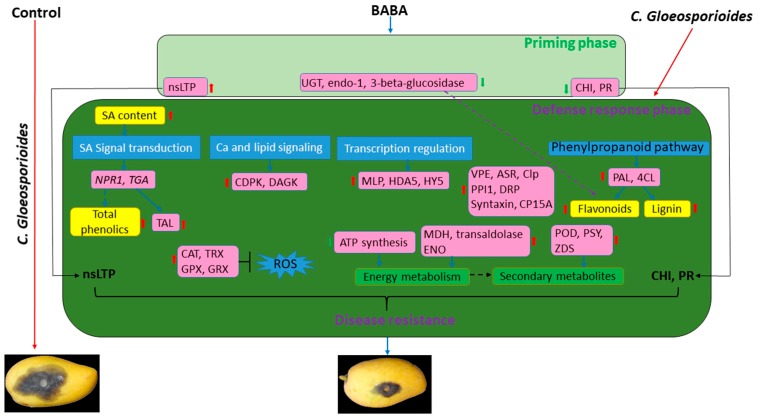
Presumptive model depicting the BABA priming defense mechanism. nsLTP: non-specific lipid-transfer protein; UGT: UDP-glycosyltransferase; CHI: chitinase; PR: Pathogenesis protein; NPR1: none expressor of pathogenesis-related genes; CDPK: calcium-dependent protein kinases; DAGK: diacylglycerol kinase; MLP: major latex protein; HDA5: histone deacetylase 5; VPE: vacuolar processing enzyme; CP15A: cysteine proteinase 15A; Clp: ATP-dependent Clp protease; ASR: ABA-stress-ripening; PPI1: proton pump-interactor 1; DRP: desiccation-related protein; PAL: phenylalanin ammonia-lyase; 4CL: 4-coumarate-CoA ligase; TAL: thaumatin-like protein; CAT: catalase; TRX: thioredoxin; GRX: glutaredoxin; GPX: glutathione peroxidase; MDH: malate dehydrogenase; ENO: enolase; POD: peroxidase; PSY: Phytoene synthase; ZDS: ζ-carotene desaturase. Red arrow indicates upregulation while green arrow indicates downregulation in BABA-treated mango fruit.

From the overview presented in Figure 8, this study provided a complete picture of the events occurring during the onset, persistence of the primed state of mango fruit. Our results assigned a clearly defined, ubiquitously applicable protein accumulation pattern to a specific stage of priming. It can be deduced that some members of nsLTP proteins were responsible for the acquisition of priming and then contributed to the maintenance of priming in response to pathogen. Meanwhile, downregulation of PR, CHI at priming stage resulted in the spring effect of BABA-induced priming defense, indicated by significant upregulation at the post-challenge stage. Additionally, BABA-induced stronger resistance of the primed fruit during the post-challenge stages was mainly attributed to the induction of antifungal factors (such as flavonoids, phenolic compounds, PR proteins, and defense-related proteins), higher activities of defense enzymes, and faster activation of defense signaling mediated by SA, Ca^2+^ signaling, MLP28, HDAC, and post-translational modification. As an energy-saving strategy, BABA priming might also alter sugar metabolism to provide more backbone for secondary metabolites biosynthesis. Importantly, PPI analysis suggested that these biological processes might contribute cooperatively to BABA-induced priming response of mango fruit to pathogen. In all, the present study outlined the acquisition and memory of BABA-primed disease defense in postharvest mango fruit.

## Supplementary Materials

The following are available online at https://www.mdpi.com/2073-4409/8/9/1029/s1, Figure S1: Venn diagrams showing the number of differentially regulated proteins in mango fruit after 4 days of storage. Figure S2: Gene expression level of SA signaling related genes, Table S1: Primer sequences of the selected genes for qRT-PCR, Table S2: Some proteins that were differentially regulated at 18 h. Table S3: Some proteins that were differentially regulated at 4 d.
