# Supplementary material for: β-Aminobutyric Acid Priming Acquisition and Defense Response of Mango Fruit to Colletotrichum gloeosporioides Infection Based on Quantitative Proteomics
Source: Cells. 2019 Sep 4;8(9):1029. doi: 10.3390/cells8091029 (PMC6770319; doi:10.3390/cells8091029)
Supplement: Supplementary file 1 [file cells-08-01029-s001.pdf]

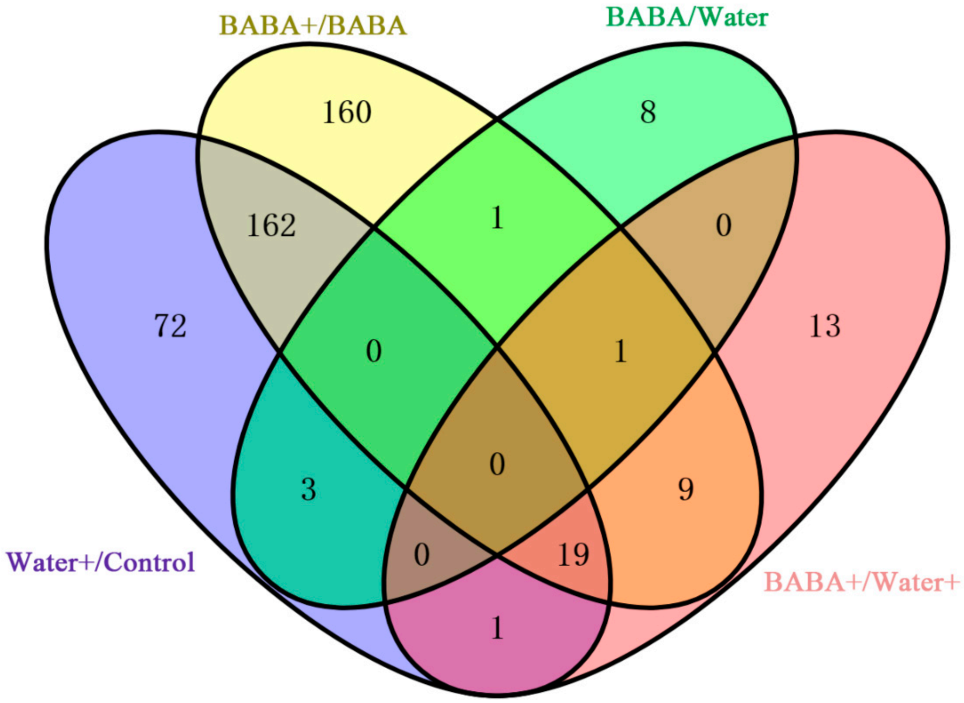

Figure S1

Venn diagrams showing the number of differentially regulated proteins in mango fruit after 4 days of storage.

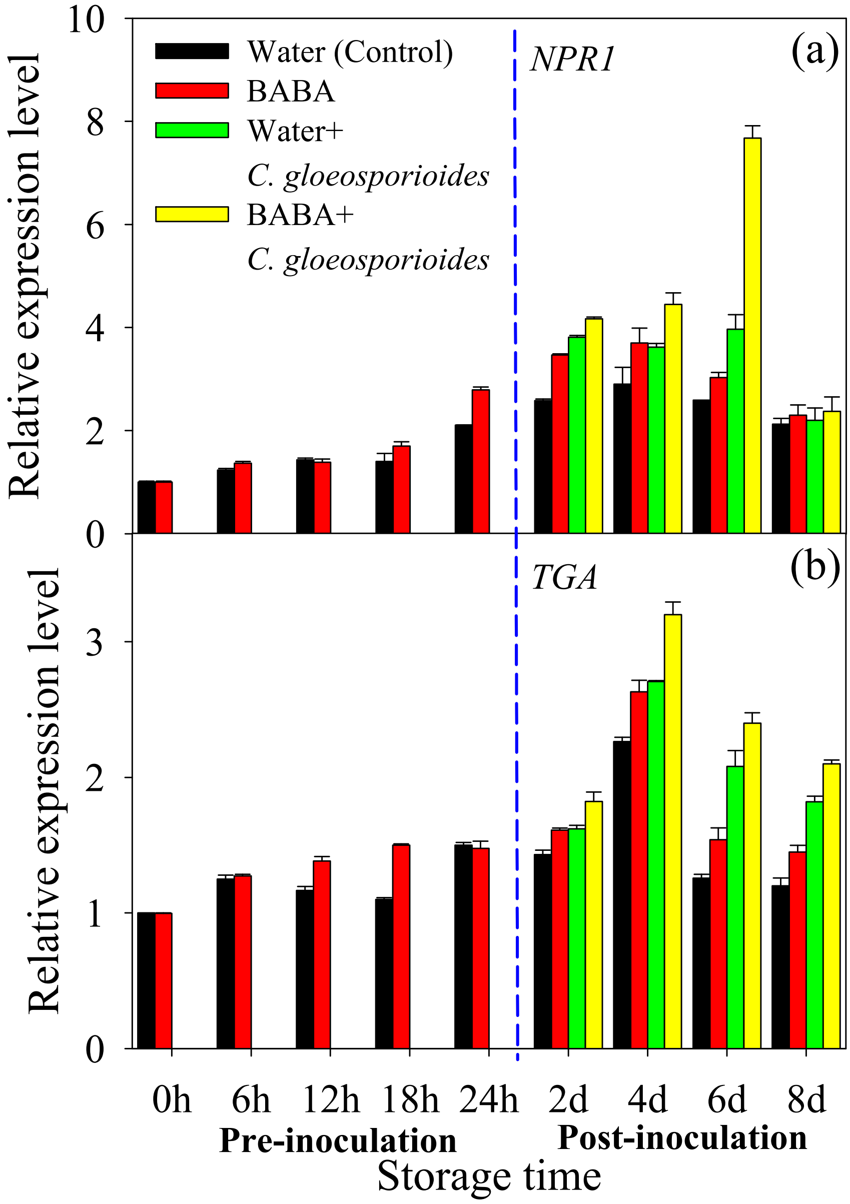

Figure S2

Gene expression level of SA signaling related genes.

Table S1. Primer sequences of the selected genes for qRT-PCR.

Gene name	Unigene ID	Forward primer (5'→3')	Reverse primer (5'→3')
MiMLP	TRINITY_DN73690_c2_g2_m.34754	ATCTGGCTCTGTCATCTGCTGGAA	TTGGCTCTGGAACCTCCTCACTC
MiPR-4	TRINITY_DN59793_c0_g1_m.27015	GACTTGCTGGCTGTGAGTGCTT	TGTGCTCCTGTCGCTGTGTTTG
MiTLP1	TRINITY_DN8225_c0_g2_m.42771	TTGTTGAGTTCACACTGGCGGAAA	TCTCGGCAGAGTCCTTCACCAC
MiHDA5	TRINITY_DN90601_c5_g11_m.61123	GCCACAAAGTCTGCCACTTGGAGAT	TGCCTTCCACCTGTCCACCTTGA

MiCHI1	TRINITY_DN17497_c0_g1_m.12170	GGTGGTGGTTCAGTTGCCAGTATT	AATGCAGCAATCTCACGCTTGGA
Mi4CL7	TRINITY_DN79018_c0_g1_m.39121	GAGGCAATACTCTGGTGGCAATGG	AGCAAGCACAATTGGAGGCACAA
MiCAD1	TRINITY_DN73359_c0_g1_m.34547	TGGTCAGCTCGGATGCAACTCA	AGAGGGTGAAACACAGGCACAGTA
MiCCR	TRINITY_DN17449_c0_g1_m.12150	TGAGACTTCGTGGACTGACCTTGA	AACACAGCACAGCTCGCATTCA
MiPOD	TRINITY_DN72550_c0_g1_m.33984	CGACGGTGTTGTTGGTGTTGGA	AACGCCAGTGTCCTCCTTACCTT
MinsLTP	TRINITY_DN107403_c0_g1_m.1949	GCTCTTGCCATTAACTGCGGTCA	AGCAGCAGCGTTAGAGTTCAATCC
MiTRX-F	TRINITY_DN60144_c1_g1_m.27160	TCGTCAGCACTGTCCTCTCCATC	TCTTGTCACCGGCGGCGTTA
MiGPX2	TRINITY_DN53600_c0_g2_m.24082	AGCGATTTGAAGTGGGTGTTTGGT	ATTGTGTAGCAGTACAGCGGGATTG
MiGS	TRINITY_DN69464_c0_g1_m.32085	GGTGGCTAATCGTGGTTGCTCAA	GACGCCGATCCTCCAAGTAACCT
MiDRP	TRINITY_DN71326_c0_g1_m.33275	ATCTTATCGGCGGATGCAGACTCT	CCAGGCTTGTGTTCATCACCAGTT
MiVPE	TRINITY_DN82192_c1_g1_m.42683	TCTATGCAACCACAGCGGCTAATG	TGTCACTGTCCTCCATCCAAGCA
MiZDS	TRINITY_DN67292_c0_g1_m.30843	TAGTTGGCGTGCCTGTTGTCACA	GCTTGCCTCAGTTGCCTTGATCG
MiENO	TRINITY_DN87366_c0_g1_m.51370	TCGTCCAAGGTCAAGTCCGTGAAG	CACACTCTTGTCGCCGTCTCTGA
MiMDH	TRINITY_DN88557_c0_g3_m.54333	GCACTGGATGGAGACGAGGATGT	CCCTGGAGGTCAGCCGAAATCAA
MiHY5	TRINITY_DN69400_c0_g1_m.32045	TGATAGCGGTTCAGTGGCAGGT	GCAACGTGGACAGTCTCTCTTCAA
MiNPR1	TRINITY_DN74852_c1_g1	CCAAGTCACCTCTTCATCCT	TCACTCTTCTTTCACTCCCC
MiTGA	TRINITY_DN70326_c0_g1	TCCAGCCGAGAAGGTCAA	CGGAGCCGCCATTTATTT
MiACTIN		ATCTGCTGGAAGGTGCTGAG	CCAAGCAGCATGAAGATCAA

Table S2. Some proteins that were differentially regulated at 18 h.

**Protein ID**	**B18h/CK0 ratio**	**Description**
**Proteins that were regulated only by BABA priming compared to Water treatment at 18 h**		
TRINITY_DN116612_c0_g1_m.4628	0.76	18.1 kDa class I heat shock protein-like

TRINITY_DN131088_c1_g1_m.8687	1.307	photosystem I reaction center subunit N
TRINITY_DN42895_c0_g3_m.20036	0.76	ribulose bisphosphate carboxylase/oxygenase activase 2
TRINITY_DN64071_c0_g2_m.29098	1.418	glucan endo-1,3-beta-glucosidase 7
TRINITY_DN67665_c1_g1_m.31054	1.301	uncharacterized protein
TRINITY_DN74169_c0_g1_m.35014	0.752	transmembrane protein 230-like
TRINITY_DN75525_c0_g1_m.36061	1.302	cathepsin B isoform X2
TRINITY_DN81561_c2_g3_m.41820	0.76	mitogen-activated protein kinase 1
TRINITY_DN85889_c1_g9_m.48371	0.744	photosystem I assembly protein Ycf4
TRINITY_DN87220_c1_g1_m.51059	0.761	pyruvate kinase isozyme G
TRINITY_DN88423_c0_g1_m.53963	0.744	putative clathrin assembly protein
TRINITY_DN88581_c2_g1_m.54400	0.755	heat shock protein 90-5
TRINITY_DN89300_c2_g1_m.56455	0.738	callose synthase 10
TRINITY_DN89756_c0_g10_m.57865	0.73	translational activator GCN1
TRINITY_DN89797_c0_g1_m.58077	0.754	kinesin-like protein KCA2 isoform X1
TRINITY_DN89974_c0_g2_m.58652	0.726	vam6/Vps39-like protein
TRINITY_DN90262_c1_g2_m.59684	0.696	UDP-glycosyltransferase 708A6-like
TRINITY_DN90262_c1_g5_m.59687	0.732	UDP-glycosyltransferase 708A6-like
TRINITY_DN90321_c1_g3_m.59938	0.69	eukaryotic translation initiation factor 3 subunit A
TRINITY_DN90623_c4_g1_m.61204	0.695	argonaute family protein
TRINITY_DN90779_c8_g5_m.62089	0.764	E3 ubiquitin-protein ligase UPL1-like
TRINITY_DN99185_c0_g1_m.64726	1.739	non-specific lipid-transfer protein 1
TRINITY_DN87915_c2_g6_m.52673	0.747	geranylgeranyl transferase type-2 subunit beta-like
**Proteins that were regulated in primed fruit relative to non-primed fruit at 18h**		
**Protein ID**	**B18h/CK18h ratio**	**Description**
TRINITY_DN17497_c0_g1_m.12170	0.609	chitinase CHI1 precursor
TRINITY_DN65762_c0_g1_m.30030	0.717	miraculin
TRINITY_DN72717_c1_g1_m.34085	0.713	pathogenesis-related protein STH-2-like
TRINITY_DN76531_c0_g1_m.36902	0.694	chalcone synthase 1-like
TRINITY_DN87189_c1_g2_m.50995	0.727	naringenin,2-oxoglutarate 3-dioxygenase

Table S3. Some proteins that were differentially regulated at 4 d.

Protein ID	BABA+/BABA Ratio	Description
**Proteins that were specifically differentially regulated in BABA-primed fruit in response to *C. gloeosporioides* infection**		
TRINITY_DN80823_c2_g1_m.41049	6.117	carbonic anhydrase 2-like
TRINITY_DN69400_c0_g1_m.32045	3.082	transcription factor HY5-like
TRINITY_DN53391_c0_g1_m.23987	2.33	biotin carboxyl carrier protein of acetyl-CoA carboxylase 2
TRINITY_DN86466_c0_g2_m.49486	2.078	fatty-acid-binding protein 3
TRINITY_DN82540_c1_g1_m.43139	2.057	SEC14 cytosolic factor-like
TRINITY_DN89949_c2_g1_m.58577	1.998	acyltransferase-like protein At1g54570, chloroplastic isoform X2
TRINITY_DN90086_c7_g1_m.59058	1.974	putative G3BP-like protein isoform X1
TRINITY_DN83008_c0_g6_m.43757	1.824	pyruvate kinase isozyme A
TRINITY_DN88098_c1_g4_m.53100	1.75	dihydrolipoyllysine-residue acetyltransferase component 4 of pyruvate dehydrogenase complex
TRINITY_DN91273_c0_g1_m.62609	1.675	6-phosphogluconate dehydrogenase, decarboxylating 1
TRINITY_DN69843_c0_g1_m.32338	1.654	fatty-acid-binding protein
TRINITY_DN87325_c0_g1_m.51284	1.646	transmembrane protein 214-B
TRINITY_DN73299_c0_g1_m.34485	1.619	uncharacterized protein LOC107174442
TRINITY_DN89679_c5_g1_m.57608	1.608	crocetin glucosyltransferase
TRINITY_DN88541_c3_g1_m.54291	1.593	probable ADP-ribosylation factor GTPase-activating protein AGD5 isoform X2
TRINITY_DN80053_c0_g3_m.40207	1.561	cysteine proteinase 15A
TRINITY_DN9041_c0_g1_m.60333	1.548	transaldolase
TRINITY_DN46934_c0_g1_m.21588	1.539	dual specificity phosphatase Cdc25
TRINITY_DN113129_c0_g1_m.3616	1.495	uncharacterized protein At2g17340-like isoform X3
TRINITY_DN107403_c0_g1_m.1949	1.493	non-specific lipid-transfer protein 1-like
TRINITY_DN90491_c0_g13_m.60622	1.491	magnesium-chelatase subunit ChlH
TRINITY_DN71326_c0_g1_m.33275	1.49	desiccation-related protein PCC13-62
TRINITY_DN83849_c0_g1_m.44914	1.489	D-aminoacyl-tRNA deacylase
TRINITY_DN56796_c2_g1_m.25585	1.483	peroxiredoxin-2E, chloroplastic
TRINITY_DN71257_c1_g1_m.33234	1.479	ENOYL-ACP REDUCTASE 1 family protein
TRINITY_DN66783_c1_g1_m.30557	1.478	cytochrome b5
TRINITY_DN18181_c0_g1_m.12323	1.47	desumoylating isopeptidase 1
TRINITY_DN83938_c0_g1_m.45088	1.469	ferredoxin--NADP reductase
TRINITY_DN86978_c0_g2_m.50492	1.463	glycosyl transferase family 2 family protein

TRINITY_DN89096_c0_g11_m.55908	1.457	peroxisomal (S)-2-hydroxy-acid oxidase-like
TRINITY_DN42853_c1_g1_m.20002	1.45	copper transport protein ATX1-like
TRINITY_DN84180_c10_g1_m.45434	1.448	3-hydroxyacyl-[acyl-carrier-protein] dehydratase FabZ-like
TRINITY_DN78900_c2_g1_m.39002	1.443	eukaryotic peptide chain release factor subunit 1-3
TRINITY_DN85387_c0_g1_m.47412	1.443	phytoene synthase
TRINITY_DN88428_c0_g1_m.53975	1.436	probable glutamyl endopeptidase, chloroplastic isoform X2
TRINITY_DN82192_c1_g1_m.42683	1.433	vacuolar-processing enzyme-like
TRINITY_DN89014_c1_g1_m.55677	1.432	proton pump-interactor 1-like
TRINITY_DN88375_c0_g1_m.53815	1.429	acyl-CoA-binding domain-containing protein 4
TRINITY_DN87366_c0_g1_m.51370	1.425	enolase 1
TRINITY_DN79018_c0_g1_m.39121	1.42	4-coumarate--CoA ligase-like 7 isoform X1
TRINITY_DN70734_c0_g1_m.32913	1.419	calcium-dependent protein kinase 21-like
TRINITY_DN115674_c0_g1_m.4242	1.413	Peroxidase superfamily protein
TRINITY_DN26309_c0_g2_m.14758	1.413	3-hydroxyacyl-[acyl-carrier-protein] dehydratase FabZ-like
TRINITY_DN7862_c0_g2_m.38713	1.413	GDSL esterase/lipase CPRD49
TRINITY_DN88557_c0_g3_m.54333	1.412	malate dehydrogenase
TRINITY_DN60144_c1_g1_m.27160	1.411	thioredoxin F-type
TRINITY_DN89203_c2_g9_m.56177	1.408	probable protein phosphatase 2C 10
TRINITY_DN54558_c0_g1_m.24460	1.407	catalase family protein
TRINITY_DN57042_c0_g2_m.25711	1.404	40S ribosomal protein S18
TRINITY_DN83035_c2_g1_m.43788	1.402	peroxisomal acyl-coenzyme A oxidase 1-like
TRINITY_DN74760_c0_g1_m.35436	1.399	protein GrpE isoform X2
TRINITY_DN75998_c0_g1_m.36473	1.399	protein RETICULATA-RELATED 1, chloroplastic
TRINITY_DN88419_c0_g1_m.53952	1.392	subtilisin-like protease SBT1.9
TRINITY_DN89396_c2_g2_m.56780	1.391	acyltransferase-like protein
TRINITY_DN76625_c2_g1_m.37007	1.39	glutaredoxin
TRINITY_DN77826_c0_g1_m.37982	1.388	3-oxoacyl-[acyl-carrier-protein] synthase II
TRINITY_DN59520_c0_g1_m.26910	1.386	subtilisin-like protease SBT3.3
TRINITY_DN86833_c0_g2_m.50198	1.384	probable ADP-ribosylation factor GTPase-activating protein AGD6
TRINITY_DN118675_c0_g1_m.5283	1.383	root FNR 2

TRINITY_DN80150_c0_g1_m.40313	1.371	mitochondrial succinate-fumarate transporter 1
TRINITY_DN83520_c2_g1_m.44432	1.371	non-specific lipid-transfer protein-like 1
TRINITY_DN87220_c1_g1_m.51059	1.371	pyruvate kinase isozyme G, chloroplastic isoform X2
TRINITY_DN87662_c0_g1_m.52088	1.37	putative uncharacterized protein DDB_G0294196
TRINITY_DN87504_c2_g4_m.51737	1.368	probable glutathione peroxidase 2
TRINITY_DN47829_c0_g1_m.22049	1.367	diacylglycerol kinase 5
TRINITY_DN84736_c0_g1_m.46261	1.367	4-hydroxyphenylpyruvate dioxygenase
TRINITY_DN85023_c0_g1_m.46752	1.366	6-phosphogluconate dehydrogenase, decarboxylating 1,
TRINITY_DN89674_c2_g1_m.57586	1.365	translocase of chloroplast 159
TRINITY_DN89611_c0_g1_m.57392	1.36	uncharacterized protein LOC18591502
TRINITY_DN60353_c0_g1_m.27273	1.355	uncharacterized oxidoreductase SSP0419
TRINITY_DN58106_c0_g1_m.26176	1.354	pectinesterase-like
TRINITY_DN84291_c0_g1_m.45577	1.351	Chloroplast ADP family protein
TRINITY_DN90176_c1_g11_m.59380	1.344	auxilin-related protein 2
TRINITY_DN82294_c0_g1_m.42827	1.343	L-ascorbate oxidase homolog
TRINITY_DN88579_c0_g2_m.54391	1.343	sulfite reductase
TRINITY_DN88966_c3_g1_m.55553	1.342	transmembrane 9 superfamily member 9-like
TRINITY_DN72781_c0_g1_m.34128	1.336	peroxisome biogenesis protein 7
TRINITY_DN78908_c1_g1_m.39005	1.334	40S ribosomal protein S14-3
TRINITY_DN90568_c9_g1_m.60994	1.333	ATP-dependent Clp protease ATP-binding subunit ClpA homolog CD4B
TRINITY_DN69975_c1_g1_m.32436	1.33	uncharacterized protein LOC102611602
TRINITY_DN74001_c0_g1_m.34921	1.327	syntaxin-121-like
TRINITY_DN67292_c0_g1_m.30843	1.326	ZDS
TRINITY_DN14999_c0_g1_m.11488	1.325	nifU-like protein 2
TRINITY_DN82527_c1_g1_m.43127	1.325	vesicle-associated protein 1-2
TRINITY_DN76971_c0_g1_m.37290	1.322	uncharacterized protein LOC104415307 isoform X2
TRINITY_DN107409_c1_g1_m.1957	1.32	mitochondrial uncoupling protein 1 isoform X2
TRINITY_DN84836_c1_g2_m.46430	1.32	acyl-coenzyme A oxidase 2, peroxisomal isoform X2
TRINITY_DN72371_c0_g1_m.33867	1.319	probable xyloglucan endotransglucosylase/hydrolase protein B isoform X1
TRINITY_DN85227_c0_g1_m.47165	1.316	alpha-mannosidase-like isoform X2
TRINITY_DN61477_c0_g1_m.27727	1.314	probable mediator of RNA polymerase II transcription subunit 37e

TRINITY_DN76958_c1_g1_m.37265	1.314	malonyl-CoA-acyl carrier protein transacylase, mitochondrial
TRINITY_DN53600_c0_g2_m.24082	1.313	probable glutathione peroxidase 2
TRINITY_DN90308_c7_g1_m.59885	1.31	AMP deaminase
TRINITY_DN89089_c1_g1_m.55892	1.309	dicarboxylate transporter 2.1
TRINITY_DN90278_c1_g10_m.59755	1.307	staphylococcal nuclease domain-containing protein 1-like
TRINITY_DN89295_c3_g1_m.56434	1.305	bet1-like protein
TRINITY_DN17332_c0_g1_m.12112	4.291	interactor of constitutive active ROPs 1
TRINITY_DN17497_c0_g1_m.12170	1.841	chitinase CHI1 precursor
TRINITY_DN57437_c1_g1_m.25912	1.373	two-on-two hemoglobin-3
TRINITY_DN77661_c1_g1_m.37825	2.036	acyl-acyl carrier protein thioesterase ATL3
TRINITY_DN78828_c0_g2_m.38930	3.059	ribulose bisphosphate carboxylase small chain clone 512-like isoform X1
TRINITY_DN88098_c1_g18_m.53097	1.678	dihydrolipoyllysine-residue acetyltransferase component 4 of pyruvate dehydrogenase complex,chloroplastic
TRINITY_DN89492_c0_g4_m.57046	1.472	uncharacterized protein C6C3.02c
TRINITY_DN90601_c5_g11_m.61123	1.567	histone deacetylase 5 isoform X1
TRINITY_DN99185_c0_g1_m.64726	1.66	non-specific lipid-transfer protein 1
TRINITY_DN88452_c0_g1_m.54040	1.538	dicarboxylate transporter 1,
TRINITY_DN73359_c0_g1_m.34547	0.769	probable cinnamyl alcohol dehydrogenase 1 [Ipomoea nil]
TRINITY_DN57158_c0_g1_m.25789	0.768	methylthioribose kinase
TRINITY_DN17449_c0_g1_m.12150	0.767	cinnamoyl-CoA reductase 1-like
TRINITY_DN17005_c0_g1_m.12020	0.766	MLP-like protein 423 [Citrus sinensis]
TRINITY_DN84746_c0_g1_m.46294	0.765	cytochrome P450 CYP82D47-like
TRINITY_DN80163_c0_g1_m.40327	0.764	probable xyloglucan endotransglucosylase/hydrolase protein 6
TRINITY_DN83794_c0_g1_m.44827	0.764	serine hydroxymethyltransferase, mitochondrial
TRINITY_DN39220_c0_g1_m.18553	0.762	non-functional NADPH-dependent codeinone reductase 2-like
TRINITY_DN88295_c1_g1_m.53568	0.762	1-aminocyclopropane-1-carboxylate oxidase homolog 1-like isoform X2
TRINITY_DN27524_c0_g1_m.15129	0.761	pyruvate decarboxylase family protein
TRINITY_DN56794_c0_g1_m.25584	0.76	light-harvesting complex I chlorophyll A/B-binding protein
TRINITY_DN47862_c0_g1_m.22064	0.759	protein EARLY RESPONSIVE TO DEHYDRATION 15

TRINITY_DN71543_c0_g1_m.33401	0.759	calvin cycle protein CP12-1
TRINITY_DN81888_c1_g1_m.42237	0.759	dual specificity protein phosphatase 12-like
TRINITY_DN71616_c0_g1_m.33445	0.751	7-deoxyloganetin glucosyltransferase-like
TRINITY_DN66487_c0_g1_m.30387	0.748	2-alkenal reductase (NADP(+)-dependent)
TRINITY_DN82234_c0_g1_m.42736	0.747	peroxisomal (S)-2-hydroxy-acid oxidase-like isoform X1
TRINITY_DN71547_c0_g1_m.33403	0.746	uncharacterized protein LOC107415849
TRINITY_DN72014_c0_g1_m.33666	0.746	protein transport protein Sec61 subunit beta-like
TRINITY_DN76200_c3_g2_m.36638	0.745	major latex allergen Hev b 5-like isoform X2
TRINITY_DN82977_c0_g1_m.43705	0.744	geranylgeranyl diphosphate reductase, chloroplastic
TRINITY_DN83278_c0_g1_m.44077	0.743	serine--glyoxylate aminotransferase
TRINITY_DN44034_c0_g1_m.20566	0.742	D-3-phosphoglycerate dehydrogenase 1
TRINITY_DN85009_c2_g2_m.46734	0.742	AtpB (chloroplast)
TRINITY_DN88262_c1_g3_m.53484	0.742	AtpA (chloroplast)
TRINITY_DN58515_c2_g1_m.26419	0.741	3-oxo-Delta(4,5)-steroid 5-beta-reductase-like
TRINITY_DN86259_c0_g1_m.49099	0.74	non-functional NADPH-dependent codeinone reductase 2-like
TRINITY_DN39371_c0_g1_m.18628	0.733	LOW QUALITY PROTEIN: photosystem II CP47 reaction center protein-like
TRINITY_DN69464_c0_g1_m.32085	0.732	glutamine synthetase leaf isozyme, chloroplastic-like
TRINITY_DN76636_c0_g1_m.37018	0.732	ATP synthase subunit delta
TRINITY_DN85027_c0_g1_m.46763	0.732	neurogenic protein mastermind
TRINITY_DN72550_c0_g1_m.33984	0.731	peroxidase 12 [Theobroma cacao]
TRINITY_DN90549_c2_g18_m.60904	0.731	photosystem II 44 kDa apoprotein
TRINITY_DN15156_c0_g1_m.11526	0.73	secoisolariciresinol dehydrogenase
TRINITY_DN53151_c1_g3_m.23878	0.728	phosphoenolpyruvate carboxylase 2
TRINITY_DN82803_c0_g1_m.43451	0.726	alkane hydroxylase MAH1-like
TRINITY_DN85009_c2_g2_m.46735	0.72	AtpE
TRINITY_DN6739_c0_g2_m.30908	0.716	glutamine synthetase nodule isozyme
TRINITY_DN72606_c1_g1_m.34013	0.716	ribulose bisphosphate carboxylase/oxygenase activase 2, chloroplastic isoform X2
TRINITY_DN90549_c2_g18_m.60906	0.714	photosystem II protein D2
TRINITY_DN37924_c0_g1_m.18098	0.713	cytochrome P450 81E8-like
TRINITY_DN90532_c2_g2_m.60804	0.713	tubulin beta-5 chain

TRINITY_DN87997_c0_g1_m.52847	0.712	LOW QUALITY PROTEIN: 65-kDa microtubule-associated protein 1-like
TRINITY_DN80063_c0_g1_m.40213	0.707	ATP synthase gamma chain
TRINITY_DN75777_c0_g1_m.36277	0.706	vestitone reductase
TRINITY_DN1009_c0_g1_m.369	0.704	galactokinase-like, partial
TRINITY_DN14477_c0_g1_m.11216	0.696	serine--glyoxylate aminotransferase
TRINITY_DN73897_c0_g1_m.34871	0.696	ribosomal RNA small subunit methyltransferase B isoform X2
TRINITY_DN87913_c1_g3_m.52658	0.696	photosystem II protein D1
TRINITY_DN73642_c0_g1_m.34724	0.69	homeobox protein knotted-1-like LET12
TRINITY_DN82941_c2_g1_m.43656	0.69	probable nucleoredoxin 1
TRINITY_DN81470_c0_g1_m.41724	0.68	GEM-like protein 5
TRINITY_DN89876_c3_g5_m.58343	0.679	protein argonaute 4-like
TRINITY_DN87913_c3_g1_m.52661	0.676	F-box/kelch-repeat protein
TRINITY_DN79990_c1_g1_m.40133	0.668	uncharacterized protein LOC18597726
TRINITY_DN22594_c0_g1_m.13753	0.665	abscisic stress-ripening protein 2-like
TRINITY_DN88369_c1_g1_m.53784	0.665	uncharacterized protein LOC102617553
TRINITY_DN47340_c0_g1_m.21834	0.644	inorganic phosphate transporter 1-4-like
TRINITY_DN30050_c0_g1_m.15940	0.643	PSI reaction center subunit II
TRINITY_DN73519_c0_g1_m.34656	0.606	random slug protein 5
TRINITY_DN47249_c0_g1_m.21785	0.598	3-ketoacyl-CoA synthase 6
TRINITY_DN133567_c0_g1_m.9673	0.583	photosystem I P700 apoprotein A1 (mitochondrion)
TRINITY_DN78951_c0_g1_m.39057	0.565	Chlorophyll a-b binding protein
TRINITY_DN64844_c0_g1_m.29477	0.551	fruit protein pKIWI501-like
TRINITY_DN47392_c0_g1_m.21858	0.546	major allergen Pru av 1

Proteins that were differentially regulated in BABA+ compared toWater+ at 4 d

**Protein ID**	**BABA+/Control+ ratio**	**Description**
TRINITY_DN107402_c5_g9_m.1948	1.445	glutathione S-transferase
TRINITY_DN115219_c0_g1_m.3996	1.631	pectate lyase 8
TRINITY_DN120408_c0_g1_m.5753	0.763	aconitase 1
TRINITY_DN123185_c0_g1_m.6356	1.325	linoleate 9S-lipoxygenase 5 isoform X1
TRINITY_DN128711_c0_g1_m.8241	1.408	ATP-dependent RNA helicase
TRINITY_DN13748_c0_g2_m.10654	1.58	ferredoxin-like
TRINITY_DN17332_c0_g1_m.12112	2.055	interactor of constitutive active ROPs 1
TRINITY_DN17497_c0_g1_m.12170	1.888	chitinase CHI1 precursor
TRINITY_DN2179_c1_g2_m.13399	2.093	tropinone reductase
TRINITY_DN42530_c0_g1_m.19874	1.319	dihydrolipoyllysine-residue acetyltransferase component 5 of pyruvate dehydrogenase
TRINITY_DN45670_c0_g2_m.21100	1.527	thaumatin-like protein 1b
TRINITY_DN57437_c1_g1_m.25912	1.307	two-on-two hemoglobin-3

TRINITY_DN59104_c1_g1_m.26704	1.301	metal tolerance protein 1
TRINITY_DN59136_c1_g1_m.26718	1.721	methionine gamma-lyase
TRINITY_DN59793_c0_g1_m.27015	1.599	pathogenesis-related protein PR-4
TRINITY_DN63773_c0_g1_m.28928	1.47	21 kDa protein
TRINITY_DN64099_c0_g1_m.29113	1.593	21 kDa protein-like
TRINITY_DN67300_c0_g1_m.30848	1.351	uncharacterized protein
TRINITY_DN71076_c0_g1_m.33143	1.347	3-glucanase family protein
TRINITY_DN72717_c1_g1_m.34085	1.396	pathogenesis-related protein STH-2-like
TRINITY_DN73690_c2_g2_m.34754	1.594	MLP-like protein 28
TRINITY_DN74586_c0_g1_m.35299	0.738	nudix hydrolase 23
TRINITY_DN77661_c1_g1_m.37825	1.437	acyl-acyl carrier protein thioesterase ATL3
TRINITY_DN78828_c0_g2_m.38930	2.439	ribulose bisphosphate carboxylase small chain clone 512-like isoform X1
TRINITY_DN80848_c0_g1_m.41073	1.476	polyphenol oxidase
TRINITY_DN81085_c2_g1_m.41339	1.689	xyloglucan endotransglucosylase/hydrolase protein 31-like
TRINITY_DN81181_c0_g1_m.41418	1.303	UPF0183 protein
TRINITY_DN8225_c0_g2_m.42771	1.531	thaumatin-like protein 1
TRINITY_DN82403_c0_g1_m.42966	1.326	splicing factor 3A subunit 2-like
TRINITY_DN82552_c0_g2_m.43165	1.422	stearoyl-[acyl-carrier-protein] 9-desaturase
TRINITY_DN85683_c1_g2_m.47945	1.641	basic endochitinase CHB4-like
TRINITY_DN85683_c1_g9_m.47946	1.387	chitinase 4-like isoform X1
TRINITY_DN88098_c1_g18_m.53097	1.388	dihydrolipoyllysine-residue acetyltransferase component 4 of pyruvate dehydrogenase
TRINITY_DN88629_c1_g5_m.54539	1.313	phosphoglycerate mutase-like protein 1 isoform X1
TRINITY_DN88838_c3_g2_m.55174	1.348	protein DMR6-LIKE OXYGENASE 2 isoform X1
TRINITY_DN89290_c0_g1_m.56412	1.317	stem-specific protein TSJT1
TRINITY_DN89492_c0_g4_m.57046	1.358	uncharacterized protein
TRINITY_DN90071_c1_g1_m.58993	1.573	L-cysteine desulfhydrase
TRINITY_DN90601_c5_g11_m.61123	1.447	histone deacetylase 5 isoform X1
TRINITY_DN99185_c0_g1_m.64726	1.422	non-specific lipid-transfer protein 1
TRINITY_DN99251_c0_g2_m.64789	1.56	glutathione S-transferase
TRINITY_DN66394_c0_g1_m.30352	1.557	expansin-B15-like
